# OncoRTT: Predicting novel oncology-related therapeutic targets using BERT embeddings and omics features

**DOI:** 10.3389/fgene.2023.1139626

**Published:** 2023-04-06

**Authors:** Maha A. Thafar, Somayah Albaradei, Mahmut Uludag, Mona Alshahrani, Takashi Gojobori, Magbubah Essack, Xin Gao

**Affiliations:** ^1^ Computer, Electrical and Mathematical Sciences and Engineering Division (CEMSE), Computational Bioscience Research Center, Computer (CBRC), King Abdullah University of Science and Technology (KAUST), Thuwal, Saudi Arabia; ^2^ College of Computers and Information Technology, Computer Science Department, Taif University, Taif, Saudi Arabia; ^3^ Faculty of Computing and Information Technology, King Abdulaziz University, Jeddah, Saudi Arabia; ^4^ National Center for Artificial Intelligence (NCAI), Saudi Data and Artificial Intelligence Authority (SDAIA), Riyadh, Saudi Arabia

**Keywords:** machine learning, sequence embedding, omics, target identification, lung cancer, colon cancer, bioinformatics, deep neural network

## Abstract

Late-stage drug development failures are usually a consequence of ineffective targets. Thus, proper target identification is needed, which may be possible using computational approaches. The reason being, effective targets have disease-relevant biological functions, and omics data unveil the proteins involved in these functions. Also, properties that favor the existence of binding between drug and target are deducible from the protein’s amino acid sequence. In this work, we developed OncoRTT, a deep learning (DL)-based method for predicting novel therapeutic targets. OncoRTT is designed to reduce suboptimal target selection by identifying novel targets based on features of known effective targets using DL approaches. First, we created the “OncologyTT” datasets, which include genes/proteins associated with ten prevalent cancer types. Then, we generated three sets of features for all genes: omics features, the proteins’ amino-acid sequence BERT embeddings, and the integrated features to train and test the DL classifiers separately. The models achieved high prediction performances in terms of area under the curve (AUC), i.e., AUC greater than 0.88 for all cancer types, with a maximum of 0.95 for leukemia. Also, OncoRTT outperformed the state-of-the-art method using their data in five out of seven cancer types commonly assessed by both methods. Furthermore, OncoRTT predicts novel therapeutic targets using new test data related to the seven cancer types. We further corroborated these results with other validation evidence using the Open Targets Platform and a case study focused on the top-10 predicted therapeutic targets for lung cancer.

## 1 Introduction

The development of novel anticancer drugs is associated with high costs, poor safety profiles, and is a time-consuming process with significant failure rates (Bhavana, 2017). Thus, several groups have proposed models developed with machine learning (ML) and deep learning (DL) techniques to address cancer-related issues. These models integrate features of the biological processes to accomplish various tasks, including identifying new gene-disease associations, pinpointing the cancer driver genes (Althubaiti et al., 2019; Althubaiti et al., 2021), predicting cancer-specific biomarkers (Pal et al., 2007; Tabl et al., 2019), predicting anticancer peptides (Arif et al., 2022), and predicting pan-cancer metastasis (Albaradei et al., 2019; Albaradei et al., 2021a; Albaradei et al., 2021b; Albaradei et al., 2022c) (Albaradei et al., 2022). There are also other models focused on cancer-related drug repurposing that predict drug response in cancer cell lines (Liu et al., 2020) and novel oncology drug-target interactions (DTIs) (Huang et al., 2016; Dezső and Ceccarelli, 2020). In addition, other groups have proposed more generic DTIs prediction methods (Thafar et al., 2020b; Thafar et al., 2020; Alshahrani et al., 2021; Alshahrani et al., 2022; Thafar et al., 2022) with high prediction performance that provides similar topic-specific information (Thafar et al., 2019). All these avenues could lead to artificial intelligence (AI) tools that support clinicians and pinpoint potential new drugs. However, the models focused on repurposing existing drugs are only useful if an effective target is known, as late-stage drug development failures are usually a consequence of ineffective targets (Harrison, 2016). Thus, identifying appropriate targets or rather disease-specific targets is one of the most crucial steps in the drug development pipeline.

In this regard, Nidhi and colleagues (Nidhi et al., 2006) were among the first groups that tried to computationally correlate experimental target fishing technologies to predict potential targets for compounds based on chemical structures alone. They trained a multiple-category Laplacian-modified naïve Bayesian model on extended-connectivity fingerprints of compounds from 964 target classes in the WOMBAT (World of Molecular BioAcTivity) chemogenomic database. As a result, they reported that the model predicted the top three most likely protein targets for all MDDR (MDL Drug Database Report) database compounds, 77% of the time (for compounds from 10 MDDR activity classes with known targets). Furthermore, the model systematically deconvolutes MDDR compounds annotated with only generic activities such as “antineoplastic” or “kinase inhibitor” to specific targets associated with the therapeutic effect, which suggests that the model can predict new targets for orphan compounds. However, since target proteins were shown to have a high degree and betweenness centrality in the human protein-protein interaction (PPI) network (Yao and Rzhetsky, 2008), Li and colleagues (Li et al., 2015) tried to address this problem in a generic manner. They constructed a PPI network and then calculated the topological feature values of proteins based on graph theory to generate feature vectors. They used the minimum redundancy - maximum relevance (mRMR) feature selection approach to select the features with discriminative information and then random forest (RF) to construct the prediction model. This study showed network-based features to be significant in scoring potential therapeutic targets (Li et al., 2015).

To the best of our knowledge, only two very recent ML methods identify novel therapeutic targets for oncology (Bazaga et al., 2020; Dezső and Ceccarelli, 2020). In the first approach, Dezso and Ceccarelli (Dezső and Ceccarelli, 2020) leveraged the growing number of large-scale human genomics and proteomics data to make *in silico* target identification. They developed an ML approach that prioritizes proteins based on similarity to targets of an approved drug. This was done by computing an extensive collection of protein features that the learning method scores based on the features’ ability to discriminate the approved target proteins from others. The model achieved high prediction performance (Area Under the Curve (AUC) of 0.89) based on an independent set of clinical trial targets. In the second approach, Bazaga and colleagues (Bazaga et al., 2020) developed a method to identify novel therapeutic targets for different cancer types. This method utilized PPI and generated latent feature vectors integrated with genomic features (gene essentiality, gene mutation, and gene expression) and tools to investigate gene-cancer associations for nine cancer types. They applied feature importance analysis and feature selection and then utilized ML classifiers to predict novel therapeutic targets for cancers. They obtained high performance for some cancers and good for others in terms of AUC (Bazaga et al., 2020).

Here, we contribute to this line of research by developing the target protein prediction method, OncoRTT, that better exploits efficient features of the known targets using more advanced approaches and integrating features from several resources to improve target protein prediction in a topic-specific manner (more importantly, specific cancer types). Thus, our method, OncoRTT, is the first attempt to use DL-based models whose primary goal is to systematically predict potential cancer-specific therapeutic targets (Thafar, 2022). The main contributions of this work can be summarized as follow.

1. We developed the first DL-based method for predicting novel cancer-type-specific therapeutic targets.
2. The DL-based method, OncoRTT, provides predictions of novel therapeutic targets per cancer type that can serve as experimental starting points for cancer-related research.
3. As a future direction, the novel therapeutic targets identified by OncoRTT will be used to establish novel oncology-related DTI predictions.
4. The side product of this work is an OncologyTT dataset, a collection of drugs and target genes associated with several cancer types, which can facilitate the development and evaluation of additional *in silico* oncology drug-target research.

## 2 Materials

This work focuses on ten globally prevalent cancer types based on the cancer burden (GLOBOCAN estimates of incidence and mortality) in 2020 (Sung et al., 2021). The cancer types included are breast, lung, colon, liver, rectum, thyroid, bladder, non-Hodgkin lymphoma, leukemia, and kidney cancers.

### 2.1 The data samples (OncologyTT dataset)

**FIGURE 1:**
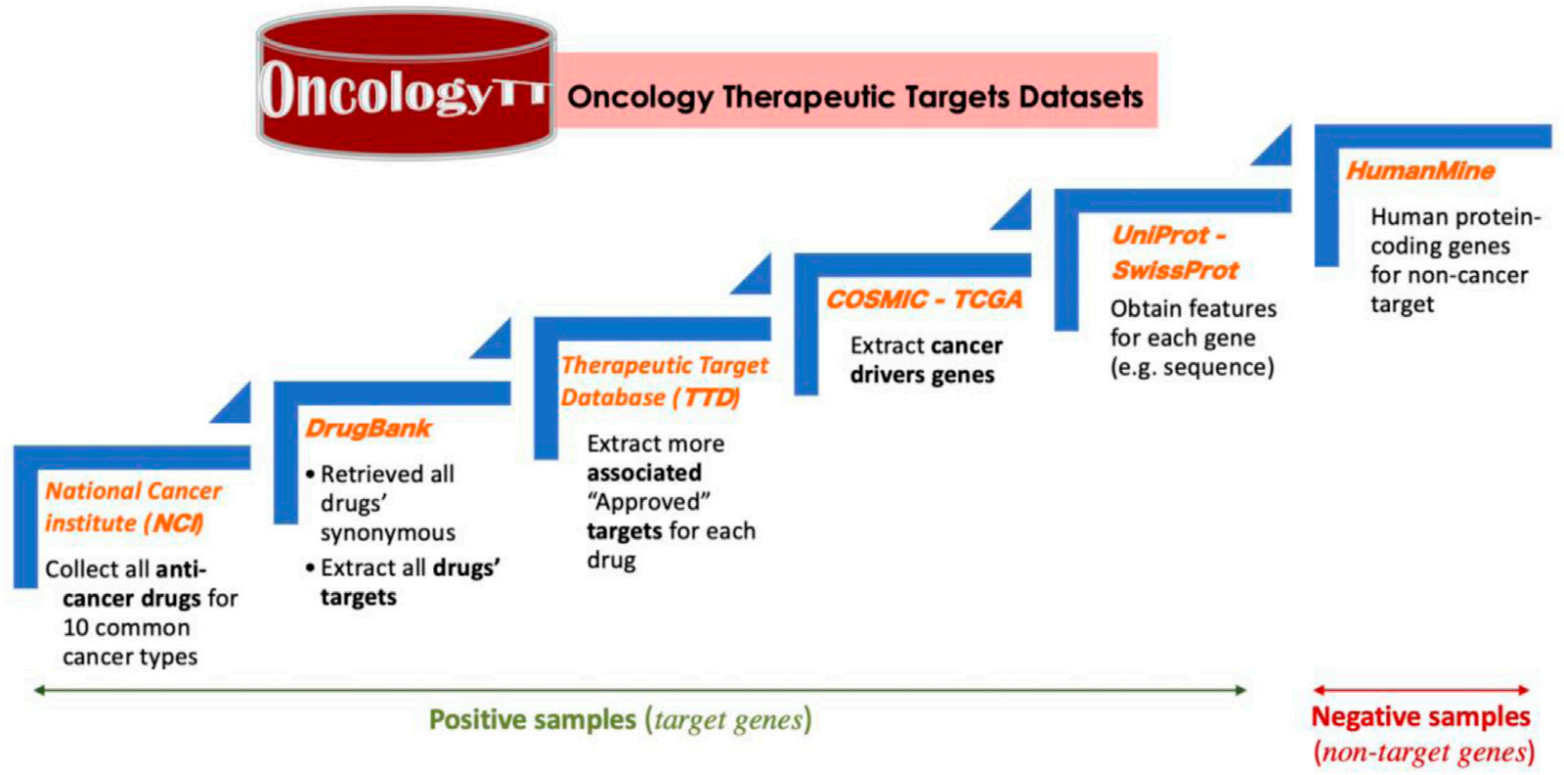
The steps implemented to create the OncologyTT dataset.

To create our data, the Oncology Therapeutic Targets (OncologyTT) dataset, several steps were applied using multiple data sources, as shown in Figure 1. OncologyTT includes drug-target information linked to ten human cancers, consisting of target and non-target samples for each cancer type (Thafar, 2022).

As a first step, we collected all anticancer drugs approved by the food and drug administration (FDA) from the national cancer institute (NCI) for each of the ten cancer types https://www.cancer.gov/about-cancer/treatment/drugs/cancer-type by July 2021. Second, for each anticancer drug, we obtained all synonyms and drug bank IDs from the DrugBank database (Wishart et al., 2008). Third, the approved targets for each drug and its synonyms (i.e., all genes with “approved” status for DTIs) were retrieved from DrugBank and the Therapeutic Target Database (TTD) (Wang et al., 2020). Since the number of target genes is limited for each cancer type and we need more data to train ML/DL models, we increased the number of targets as a fourth step. We applied this step by adding biomarker genes that are significantly overexpressed (Bao et al., 2019; Morani et al., 2021) from the complete gene expression (GE) in the Catalogue Of Somatic Mutations in Cancer (COSMIC) database https://cancer.sanger.ac.uk/cosmic/download. COSMIC is the world’s largest and most comprehensive resource for exploring the impact of somatic mutations in the human cancer (Bamford et al., 2004). It also provides all gene expression level 3 data from The Cancer Genome Atlas (TCGA) database (Weinstein et al., 2013; Ganini et al., 2021) portal for the current release (we downloaded it in August 2021). Our reasoning is that biomarker genes (also called tumor markers) can provide indications about cancer, which means they form part of the key cancer-related modules, making them candidate targets (Henry and Hayes, 2012; Kamel and Al-Amodi, 2017). The details of how we collected the biomarker genes that are significantly overexpressed are provided in Supplementary Material Section 1. Finally, using the UniProt web server (UniProt, 2021), we refined all the target genes by removing all the genes with no SwissProt records (i.e., records that are manually annotated and reviewed with information extracted from literature or curator-evaluated computational analysis). At the end of this process, we obtained all the positive genes for our dataset with SwissProt records in the UniProt database.

We additionally generated a negative dataset (i.e., non-target genes) for our classification model. First, we obtained a pool of human genes by retrieving the gene name and protein name for every gene entry in HumanMine www.humanmine.org, a segment of the InterMine project (Smith et al., 2012), that serves as an integrated warehouse of *Homo sapiens* genomic data. Then, we removed all the positive gene set entries from the pool of human genes. As a result, we have a negative dataset of more than 17,000 human protein-coding genes. Finally, we randomly retrieved the negative samples (non-target genes) from this pool for each cancer type (without any overlap of the protein-coding genes between the cancer types), equal to the number of positive samples. That is, even if the negative set is chosen randomly, there may be some biases in the dataset, therefore we created random negative datasets per cancer type to ensure the potential biases do not affect all the tests. It is worth mentioning that we have generated the same number of negative samples as the positive samples to obtain a balanced dataset, which is important in the ML classification problem to give equal priority to each class and avoid poor predictive performance for the minority class or biased classification.

All the steps mentioned above allowed us to obtain the final dataset, “OncologyTT”, which includes the positive gene dataset (i.e., target) and the negative gene dataset (i.e., non-target) summarized in Figure 1.

For all positive and negative samples (i.e., target and non-target genes) in our datasets, the amino-acid sequences were downloaded in August 2021 from the Uniprot database (UniProt, 2021) using the primary gene name. Also, we obtained all the UniProt IDs and the protein names. Table 1 summarizes this dataset categorized based on the ten cancer types. The total number of all data samples (Genes) with no duplicates is 3,117. Briefly, for each cancer type, we provide the number of anticancer drugs with at least one approved interaction (in column 2), the number of targets that interacted with drugs (in column 3), the number of unique targets that interacted with approved anticancer drugs with no duplicate (as multiple drugs can interact with the same target protein) (in column 4). The fifth column indicates the number of over-regulated cancer driver genes we consider positive samples. The sixth column is a sum of the fourth and fifth columns indicating the total number of positive genes, while the next column is the total number of negative genes.

**TABLE 1:** OncologyTT dataset statistics for the ten cancer types. Each cancer type includes the number of anticancer drugs, targets that interacted with drugs, the unique targets with no duplicates, over-regulated cancer driver genes, and the total number of positive and negative genes.

Cancer type	# Of the approved drugs	# Of DTIs	# Of approved targets	Cancer genes	Total positive genes	Negative genes	All genes
1- Bladder	13	28	24	91	115	115	230
2- Breast	40	106	71	92	163	163	326
3- Colon	16	53	39	89	128	130	258
4- Kidney	19	72	43	100	143	143	286
5- Leukemia	54	188	134	81	215	215	430
6- Liver	10	59	34	100	134	134	268
7- Lung	39	110	68	90	158	158	316
8- non-Hodgkin’s lymphoma	47	116	92	81	173	173	346
9- Rectal	16	53	39	81	120	120	240
10- Thyroid	10	58	33	100	133	133	266

### 2.2 The Cancer-Target dataset used by the baseline method

We downloaded the Cancer-Target datasets on 20 September 2021, from the GitHub link: https://github.com/storm-therapeutics/CancerTargetPrediction/tree/master/analyses_data/training_sets_genes. This dataset consists of target and non-target genes for nine common cancers. However, we excluded two cancer types not included in our study. Table 2 provides the statistics of this dataset (Bazaga et al., 2020). In our work, we utilized this dataset for two purposes. First, we used it to perform a fair comparison with the baseline method by using their dataset and following their experimental settings for training and testing, explained later in Section 4.2. Second, we used it as unseen independent test data (new genes not part of the training stage). We considered all random negative samples initially generated from the pool of human genes as unlabeled data samples and then predicted the novel therapeutic targets. We applied our methods’ pipeline for feature extraction to this dataset, including omics features and BERT embedding features.

**TABLE 2:** Statistics of the Cancer-Target baseline methods’ datasets. It includes the number of targets, the number of cancer genes, and the total number of positive and negative genes for each of the seven cancer types.

Items	Bladder	Breast	Colon	Kidney	Leukemia	Liver	Lung
Target genes	26	58	32	31	99	26	11
Cancer genes	13	36	61	1	203	1	63
Total number of positive genes (target + cancer genes), excluding genes with no data available	39	87	83	32	228	27	67
The number of negative genes used for each set	39	87	83	32	228	27	67

It is worth mentioning, we considered all random negative samples initially generated from the pool of human genes as unlabeled data samples and then predicted the novel therapeutic targets. This justification is based on a study (Bekker and Davis, 2020) that shows unlabeled data, which may include both positive and negative samples, can be used as the learning process can be done through positive samples, called positive learning or PU learning. The difference between PU learning and regular binary classification is that during the training, only some of the positive samples in the training data are labeled, but none of the negative samples are. PU has attracted increasing interest within the ML methods as this type of data naturally appears in several application areas, including target identification.

## 3 Methods

### 3.1 Problem formulation

This study describes the goal of identifying the therapeutic targets as a binary classification problem. As mentioned in the previous section, we generated all data samples (i.e., human genes) in our datasets that can be represented as vector *X = {x*_*1*_ *, x*_*2*_ *, …, x*_*n*_ *}* where n is the number of all data samples. Since our problem is supervised learning, we also provided all data samples with their class labels *Y = {y*_*1*_ *, y*_*2*_ *, …, y*_*n*_ *}* by specifying if the cancer gene is a target (i.e., positive samples) or if it is non-target (i.e., negative samples) such as:

$$yi=\left\{0,x_{i}\;is\;non-target:1,x_{i}\;is\;target\;gene\right\}$$ (1)

We followed the same methodology for all cancer types. For each data sample (gene), we extracted different features from multiple resources, as explained later. The classification model aims to find the hidden patterns and associations between genes and their labels based on the feature vector (FV) and then predict the class labels (i.e., target or non-target).

### 3.2 OncoRTT model workflow

**FIGURE 2:**
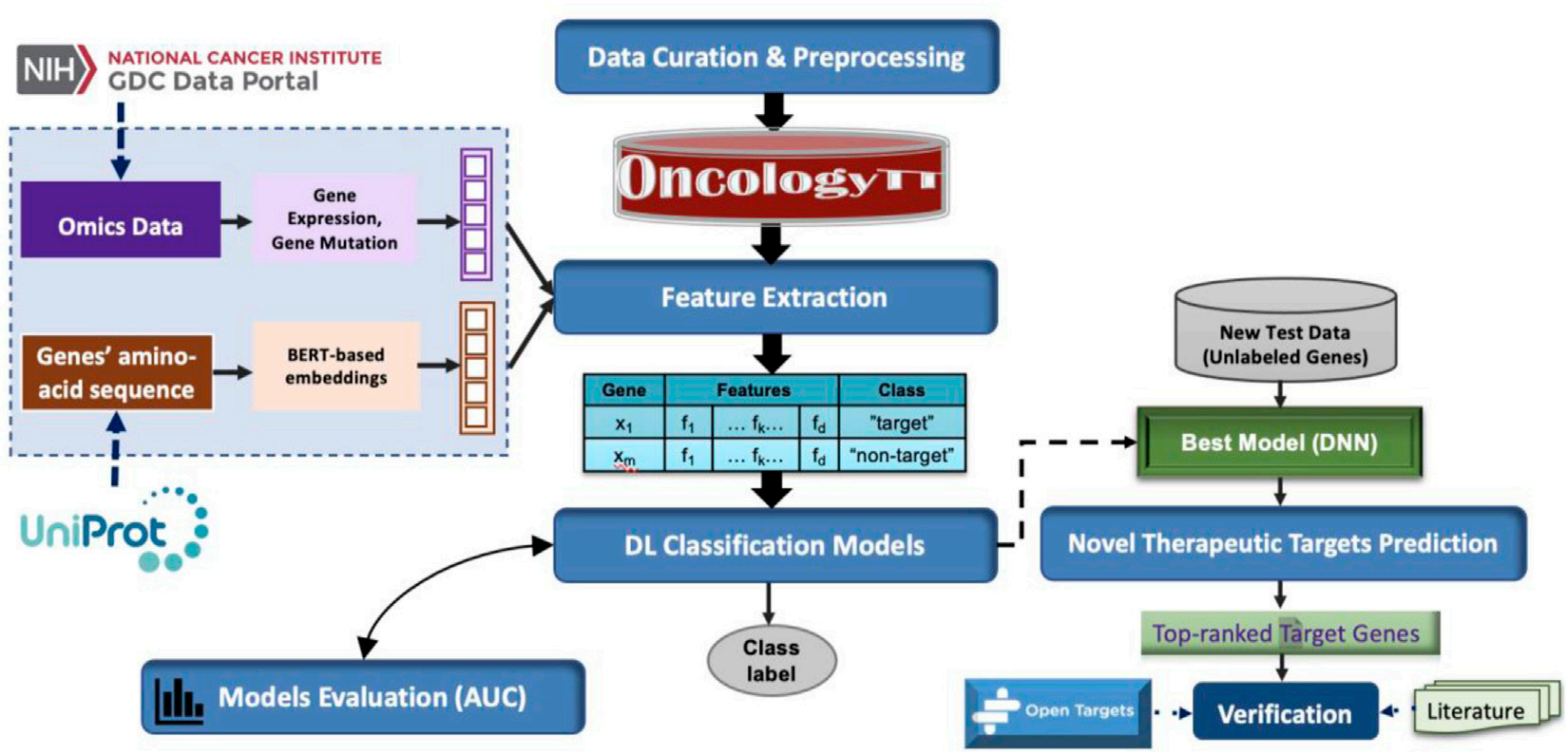
The workflow used to build the OncoRTT model consists of six main steps.

Figure 2 provides the workflow used to develop the OncoRTT model, which comprises six main steps applied to each cancer type separately. These steps are summarized as follows.

1. Generating data samples (consisting of target genes and non-target genes),
2. Extracting and integrating features from amino-acid sequences by applying BERT-based embeddings and from omics features,
3. Building several classifiers for target prediction,
4. Retraining the best-performing DL model using the whole dataset,
5. Utilizing new independent test data to predict novel therapeutic targets,
6. Validating the novel therapeutic target using multiple sources.

We provide a detailed explanation of each step below.

### 3.3 Feature extraction

The amino acid sequences are the fundamental building blocks of proteins, and the amino acids’ chemical and chemo-physical properties define the protein’s biological activity, specifically, the drugs that bind to it. Thus, a major Bioinformatics objective is to analyze amino-acid sequences of proteins and infer different structural and functional information (UniProt, 2021). Therefore, amino acid sequences have been used as the main source for features to develop several methods to predict if a protein is a target (Bakheet and Doig, 2009; Wang et al., 2014; Bull and Doig, 2015; Kumari et al., 2015; Kim et al., 2017), to predict the proteins’ binding sites (Yan et al., 2006; Andrews and Hu, 2015), or to predict proteins functionality (Kulmanov et al., 2018; Kulmanov and Hoehndorf, 2021; Sara et al., 2021). This indicates the importance of amino-acid sequences. Another advantage of the amino acid sequences is that they are known and available for all proteins (i.e., genes) in contrast to other types of information that are not available for all proteins, such as proteins’ interactions with other proteins or drugs.

Additionally, single and multi-omics data have been widely used to predict potential target proteins (Ferrero et al., 2017; Ding et al., 2018; Liang et al., 2019; Bazaga et al., 2020; Nicora et al., 2020). Omics technologies enable screening biological samples to search for novel targets (Paananen and Fortino, 2020). Genome-wide association studies are crucial for new drug targets’ prediction or validation. This type of study can provide a systematic strategy to evaluate the drug targets’ therapeutic efficacy and related side effects and understand their action mechanisms (Matthews et al., 2016). Thus, we extracted our essential features from amino-acid sequences as the primary source to differentiate between the target and non-target proteins. We also extracted omics data as a secondary source of features that can potentially complement or enhance the prediction of target and non-target proteins (we provide the prediction performance using the embedding method with and without the omics data in the Results and Discussion section). The subsequent subsection describes the feature extraction process in more detail.

#### 3.3.1 Sequence embedding-based features

Bidirectional Encoder Representations from Transformer (BERT) is a well-known DL-based embedding method that has many benefits over conventional sequential models such as Long Short-Term Memory (LSTM) and Gated Recurrent Unit (GRU). It has promising achievements in several natural language processing (NLP) tasks by extracting data patterns using unsupervised learning from massive unlabeled data. BERT-based models have been used in the biomedical domain (Zhang et al., 2019; Sun et al., 2021). Furthermore, the BERT technique has been applied to protein’s amino acid sequences to generate crucial feature representations used in different downstream tasks (Ali Shah et al., 2021; Ali Shah and Ou, 2021; Charoenkwan et al., 2021). Thus, we applied a BERT-based embedding model (Vaswani et al., 2017; Devlin et al., 2018) called ProtTrans (Elnaggar et al., 2022) to automatically extract crucial features from the amino-acid sequences that capture the most significant properties for each gene in our dataset. The ProtTrans models are state-of-the-art pre-trained models for proteins (Dallago et al., 2021), trained on thousands of GPUs from Summit and hundreds of Google TPUs using various Transformers Models.

In this work, we used one of the ProtTrans models called ProtBert-BFD (Elnaggar et al., 2022). ProtBert-BFD was trained on the BFD-100 dataset (Steinegger and Söding, 2018; Steinegger et al., 2019) containing up to 2,122 million protein sequences for the protein language model (LM). It was trained for 800 k steps for sequences with a max length of 512 and an additional 200 k steps for sequences with a max length of 2000, enabling the model to first extract crucial features from shorter sequences and then perform the training on the longer sequences more efficiently.

The ProtBert-BFD model that we used consists of several layers: 30, hidden layers size: 1,024, hidden layers intermediate size: 4096, number of neurons: 128, masking probability: 15%, optimizer: lamb (You et al., 2019), learning rate: 0.002, weight decay: 0.01, and the number of all tuned parameters: 420M. Please refer to the main ProtTrans paper (Elnaggar et al., 2022) for more details.

We directly used the pre-trained ProtBert-BFD model in a transfer-learning fashion that can be used for different downstream ML tasks, predicting targets/non-targets in our case. We applied the ProtBert-BFD model on our dataset genes’ amino-acid sequences per cancer type to embed vector representations per sequence. Therefore, we automatically extracted the information learned by the protein LMs through embeddings (i.e., biological sequence representations from the last hidden state of the protein LM) with a size equal to n*1,024, where n is the number of genes in each cancer type, and 1,024 is the dimension of the embeddings. We used these embeddings integrated with other features as input to the DL classifiers.

#### 3.3.2 OMICS-based features

For the omics data, we used TCGAbiolinks (Colaprico et al., 2016; Mounir et al., 2019), a R/Bioconductor package that provided an application programming interface (API) to access, download, and prepare data from the GDC platform and TCGA data for analysis. We used the TCGAbiolinks package to access ten TCGA projects for ten cancers we work with to obtain data for primary tumors but using different tissue/disease types. Also, we used this package to preprocess the legacy (hg19) or harmonized (hg38) TCGA datasets. We accessed and downloaded the required data in October 2021. Supplementary Table S1 provides the details of each cancer, including the project name, the name of the study, and the tissue type. First, we defined a list of ten samples for each cancer by providing the relative TCGA barcodes for the query. Then we defined a list of genes that appears in these samples to collect the omics features, i.e., the expression levels and mutations associated with every gene. However, the number of samples decreases when we remove some samples with no gene expression or mutation data available, as is the case for Rectal cancer. To obtain gene expression data, we specified the platform as “Illumina HiSeq” in the gene expression category GDC-query function. For gene mutation, we used “add.gistic2. mut” in the GDC-prepare function that indicates if a list of genes is given, columns with gistic2 results from GDAC firehose (hg19), and a column indicating if there is a mutation in that gene or not by giving values of TRUE or FALSE. These values are saved in MAF (mutation annotation formats) files to get each gene and its mutation information.

After we obtained the expression and mutation data for all the genes, we extracted features for each gene across several patient samples for matching cancer types. Since our data samples are the genes, not the patients, we aggregated gene expression values for each gene by finding the maximum, average, median, and minimum expression values over all patient samples for corresponding cancer. Therefore, each gene is represented by four features representative of gene expression level across several patient samples, which also capture whether or not the gene is always highly expressed or not. Furthermore, we calculated the gene mutation feature by counting how many times each gene is mutated across all patient samples used per cancer type. Finally, all features are combined and then normalized using min-max normalization.

### 3.4 Classification model for prediction

After completing the feature extraction process and obtaining a feature vector (FV) for all genes per cancer, we fed the three sets of FV into the classifiers, which include two ensemble ML classifiers, one classical ML classifier, and one DL classifier, to predict target/non-target genes, as illustrated in Figure 2. We implemented RF, eXtreme Gradient Boosting (XGBoost), and support vector machine (SVM) classifiers using Scikit-learn (Pedregosa et al., 2011) or XGBoost (Chen et al., 2019) libraries, respectively, for target identification. The DL model we utilized is a deep neural network (DNN). DNN performed better than the ML classifiers in all experiments when using the Integrated-based FV and Embedding-based FV for all cancer types but performed the worst when using only Omics-based FV, with only five features. This result may be a consequence of DL models working better with larger feature numbers. We report the DNN results and provide the prediction performances for RF, XGBoost, and the SVM classifiers in Supplementary Material Section 2. To improve the results, we optimized multiple parameters for the DNN while keeping some default values for other parameters during the training stage using only training data to evaluate several configurations and then selected the model with the best configurations. We applied the same classifier’s configuration to all cancer types. After that, the evaluation was performed using the test data. We implemented the DNN using Python Keras (Chollet and others, 2018) with the TensorFlow backend. Table 3 provides the most critical parameters tuned for the DNN classifier with selected values, and Table 4 provides the architecture of the proposed DNN model.

**TABLE 3:** The optimized parameters with multiple tested values. Bold font indicates the selected value for each parameter.

Parameters	Tested values
Node size in the hidden layers	[8, 12, 16, **32, 64**]
Activation function	[ **‘tanh’**, ‘relu’, ‘sigmoid']
Optimizers	['SGD, ‘Adam, **‘Nadam'**]
Batch size	[4, 8, **16**, 32]
Number of epochs	[10, 15, **20**, **30**,50,100]
Learning rate	[0.1, **0.01,** 0.001]

**TABLE 4:** The proposed DNN model’s architecture with each layer’s parameters.

DNN architecture component	Parameters
The input layers (3 different sets of features)	**1029**-dimension FV: Integrated-based features: (1,024 embeddings +5 OMICS features)
	**1024**-dimension FV: Embeddings-based features
	**5**-dimension FV: OMICS-based features
The hidden layer1 - Dense layer	neurons = 64, activation = ‘tanh’, kernel_initializer = ‘normal’
	kernel_regularizer = ‘l2’, bias_regularizer = ‘l2′
The hidden layer2 - Dense layer	neurons = 32, activation = ‘tanh’
	kernel_regularizer = ‘l2’, bias_regularizer = ‘l2′
The output layer	neurons = 1, activation = ‘sigmoid’
The Compiler	loss = 'binary_crossentropy’, optimizer = 'nadam’, metrics = 'accuracy'

### 3.5 Evaluation protocols

This section introduces the evaluation metrics we used to measure the accuracy of our prediction method and the experimental settings. Table 1 provides the number of positive and negative samples per cancer type, which reflects that our dataset is balanced. Thus, to evaluate our model’s prediction performance, the area under the receiver operating characteristic (ROC) curve (AUC) (Davis and Goadrich, 2006) is calculated. To obtain the AUC, we first calculated the false positive rate (FPR) and true positive rate (TPR) (also called recall or sensitivity) (Powers, 2011), based on true positive (TP), false positive (FP), true negative (TN) and false-negative (FN) values, as shown in Eqs 2, 3, respectively. Then, the ROC curve is constructed using different TPR and FPR values of different thresholds to calculate the AUC. The closer the value of AUC is to one, the better the performance is. We have selected the AUC metric to better assess our model performance and show its robustness. When AUC is high, it illustrates that the FP is low, and with no high false-positive prediction problem.

$$FPR=FP/\;\left(TN+FP\right)$$ (2)

$$TPR=TP/\left(TP+FN\right)$$ (3)

For the experimental setting we implemented to evaluate the OncoRTT methods’ prediction performance and robustness, we independently applied stratified 10-fold cross-validation (CV) on each cancer dataset. Therefore, the data were randomly split into ten subsets in a stratified way where each subset must include the same percentage of the target and non-target genes (i.e., negative and positive samples). Then, we held one subset for testing and used the remaining nine subsets to train the model. This process was repeated ten times to have each subset of the data in the test data. Finally, we averaged the AUC that is calculated for each fold for all ten folds.

Furthermore, another evaluation setting used by (Bazaga et al., 2020), was also implemented to compare our results with this baseline method using their dataset and their procedure to perform a fair comparison, which is explained in more detail in the comparison section. Finally, we retrained our models using all positive and negative data samples in our datasets and then applied these models on new unseen test data to predict the labels of this new data (i.e., predict the novel therapeutic targets as positive data).

​​We ran all experiments on a Linux Ubuntu 18.04.5 LTS Intel Xeon Platinum 8176 workstation, 64-bit OS, with 112 processors and two GPUs: Quadro and Titan, with CUDA version 11.0. For implementation, we mainly used Python version 3.8, and we used R version 4.1.1 for parts related to omics features preprocessing, and gene expression and mutation features.

## 4 Results and Discussion

We systematically evaluated the OncoRTT method’s performance using the datasets we created. Next, we compared the performance of our method with the state-of-the-art method. Finally, we predicted the novel target genes using unseen independent test data.

### 4.1 OncoRTT prediction performance

**FIGURE 3:**
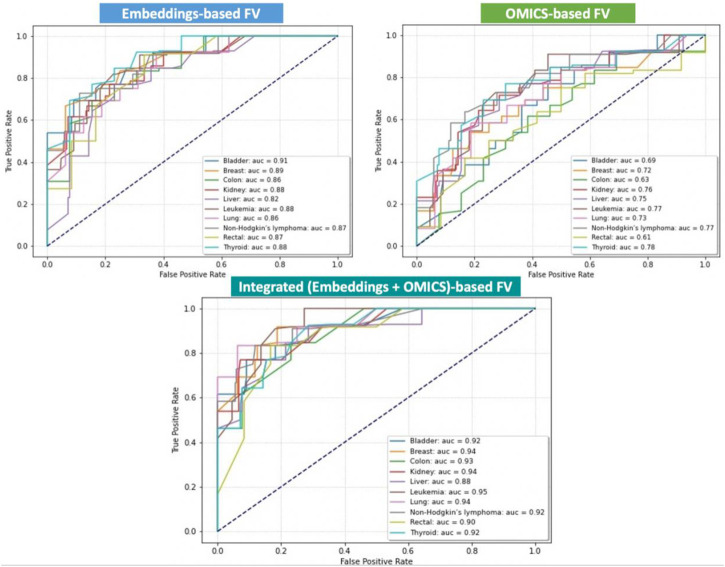
Prediction performances of the DL-based OncoRTT model using three different sets of FV for 10 cancer types in terms of AUC. The dashed line is the null model (AUC = 0.50).

We aim to integrate the omics features extracted from the expression and mutations associated with each gene with the BERT embedding features automatically generated from the amino-acid sequences and use them for training, testing, and evaluating our method. However, to show the effectiveness of the feature integration process, we also obtained results using the omics features as stand-alone and the BERT embedding feature as stand-alone. Thus, we separately trained and tested our proposed DNN using three distinct sets of FVs: OMICS FV, BERT-Embeddings FV, and Integrated FV for each cancer type separately. In addition, we quantitatively evaluated three versions of OncoRTT models in terms of AUC calculated as the average performance of models on the test set during the 10-fold CV. Figure 3 shows all obtained results using the three distinct sets of FV for ten cancer types in terms of AUC. The results exhibit consistency with regard to achieving superior performance when using the integrated FVs, the second-best performance when using the BERT-Embeddings FVs, and the worst performance when only using OMICS FVs across all cancer types.

Using the different features for several cancer types highlighted a few key aspects worth mentioning.

- The best results were obtained when we used the leukemia data (AUC = 0.95) for the integration model, and the results obtained for the other two models (Embeddings-based FV and OMICS-based FV) were also among the highest (88% and 77%) compared to the other cancers for each model. The reason may be the leukemia dataset is larger than the other datasets collected, and DL models generally perform better with more extensive data.
- Using the OMICS FVs alone achieved the worst performances for all the cancer types, which is expected as only a small number of features (only five features) were included. However, combining the OMICS FV with the BERT-Embeddings FVs across all cancer types significantly improved the OncoRTT prediction performances. That is, the result shows the contribution of the BERT-Embeddings FVs translates into a range of gain of about 13% (which is a substantial increase in the case for liver cancer) up to 29% (rectal) and 30% (colon) in the different cancer types. This result shows that the BERT-Embeddings FVs contributed substantially to the overall prediction achieved using the Integrated FVs.
- On the other hand, using the BERT-Embeddings FVs alone achieved much higher performances for all the cancer types, but the performances were still lower than the prediction performances achieved when using the Integrated FVs. Thus, despite this low (AUC <0.70) or acceptable (AUC >0.75) performance in some cases, when we only used the OMICS FVs, when we combined the BERT-Embeddings FVs with the OMICS FVs across all cancer types, the result shows the contribution of the OMICS FV that translates into a range of gain of about 1% (which is a very slight increase as is the case for bladder cancer) up to 7% (colon, leukemia) and 8% (lung cancer) for the different cancer types. The OMICS FVs enhanced the prediction performance because therapeutic targets are often the mutated or overexpressed genes underlying the cancers’ progression (Gibbs, 2000). Overall, these results show that both the BERT-Embeddings FVs and OMICS FVs contribute substantially to the high prediction performances achieved with the Integrated FVs.
- Even though the Integrated FV produced the best-performing models, the OncoRTT method achieved promising results for all cancer types when we fed only the BERT-Embeddings FVs to the DNN classifier (0.92 > AUC >=0.82). These results demonstrate the effectiveness of DL in two aspects: 1) The feature extracted by the ProtBert-BFD DL-based model revealed that the LM-feature representation (i.e., embeddings) from unlabeled and complex biomedical data represented in the protein sequences captured critical biophysical and biological features of the protein. 2) The prediction, where the DL method can identify the hidden pattern from the embeddings and better distinguish the target genes from non-target genes by giving the features different weights based on their importance and using them in the prediction process. However, it is not an easy task to interpret these auto-generated features (Ching et al., 2018).

Finally, to show the robustness of our DL models and verify that the high performance of our method in all cancer types was not random, we implemented the Y-Randomization test (also called Y-Permutation) (Rücker et al., 2007). The Y-randomization test is a non-parametric statistical approach to validate the quantitative structure-activity relationship (QSAR) models. To perform this test, we compare the DL model performance trained using the original dataset *versus* several versions of our DL model trained using the shuffled datasets. Therefore, for each cancer type separately, we first trained the DL model using the original data (i.e., FVs and labels) and obtained the results using the test data. After that, for 100 iterations, we fixed the FVs but scrambled the labels, trained the model over the new features-labels pairs, and acquired the new performances. The evaluations have been done using test data in terms of the R-squared (R^2^) evaluation metric, which is commonly used to measure the goodness of fit (Rücker et al., 2007). Consequently, we proved that OncoRTT prediction performance is statistically significant with the probability values (*p*-values) < 0.05 for each cancer type as shown in Table 5 compared to 100 randomized DL model results that were not statistically significant with *p*-values >= 0.05. Moreover, OncoRTT DL models obtained good R^2^ results for all cancer types compared to the 100 DL models trained using the shuffled datasets, obtaining low (<=0.50) or negative R^2^ results. Negative R^2^ implies that the model does not observe the data trend leading to a worse fit than the horizontal line (i.e., picked by chance), which means poor prediction performance as expected for models trained using shuffled data. Getting very low *p*-values provides evidence of a dependency between the features and the labels, and our DL model unveiled these correlations and patterns.

**TABLE 5:** The *p*-values and R^2^ evaluation metrics for all cancer types that demonstrate the DL models prediction performance is statistical significance.

Cancer type	Original DL models	
	*p*-value	R^2^
Bladder	3.9e-07	0.85
Breast	0.0012	0.96
Colon	7.7e-05	0.92
Kidney	2.9e-06	0.93
Liver	5.5e-05	0.62
Leukemia	0.0004	0.66
Lung	2.1e-06	0.96
Non-Hodgkin Lymph	0.0061	0.85
Rectal	7.3e-07	0.95
Thyroid	0.0052	0.71

### 4.2 Comparison with the baseline method

To illustrate the OncoRTT method’s effectiveness, we compared it with the previous work (Bazaga et al., 2020), which is, as far as we know, the only work focused on predicting therapeutic targets for specific cancer types making it the state-of-the-art method. Therefore, to provide a decent comparison of prediction performances, we used the same datasets (Bazaga et al., 2020) created by this method, followed the same experimental setting, utilized the same evaluation metrics, and used the optimal parameters results provided by them.

**FIGURE 4:**
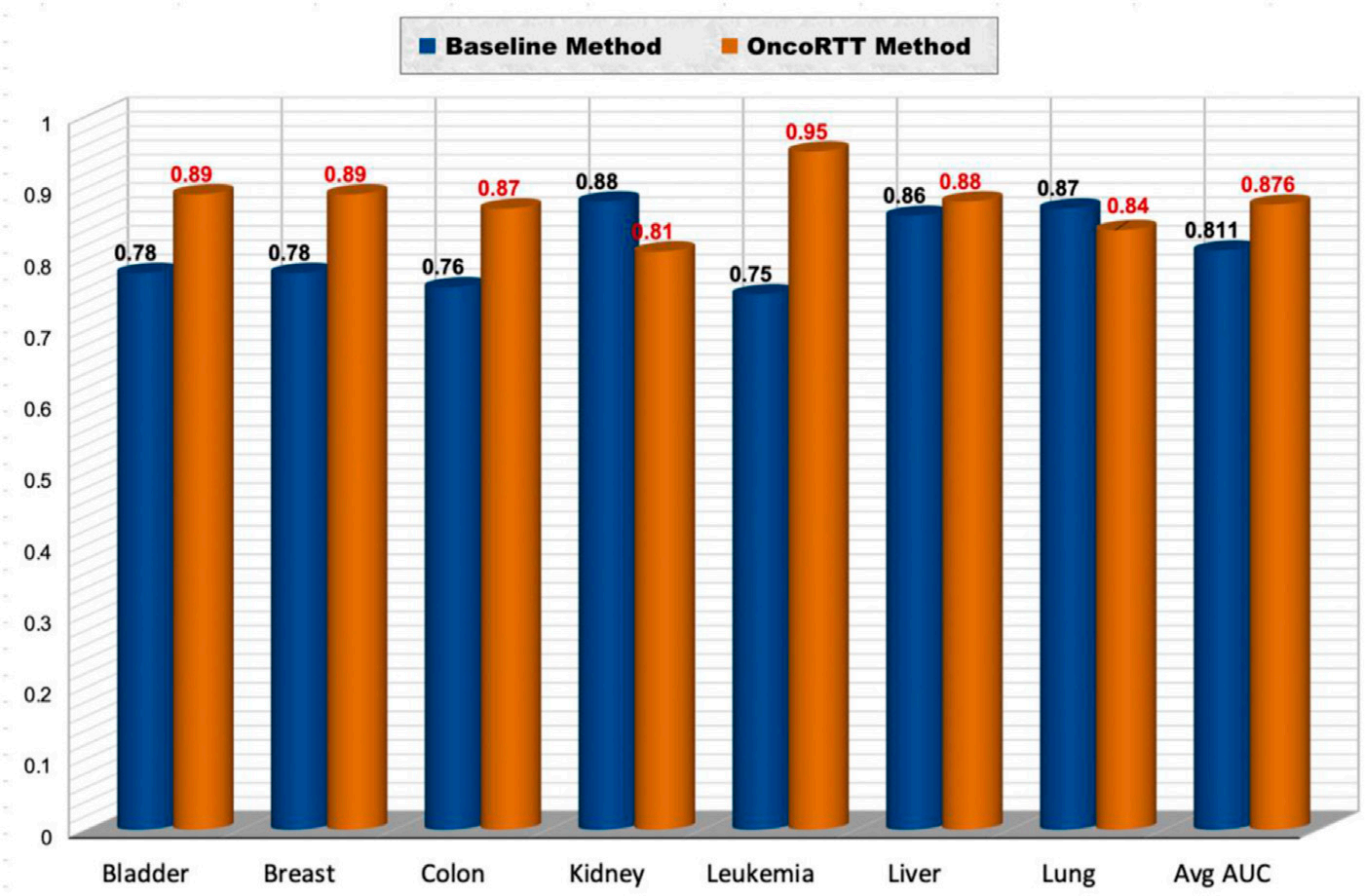
Performance comparison of the OncoRTT method vs. the baseline method in terms of AUC and average AUC for seven cancer types.

Using this dataset (Bazaga et al., 2020), we repeated our feature extraction steps for each cancer that belongs to the seven shared cancer types. Thus, we first generated BERT embeddings for all gene sequences using the ProtBert-BFD model in each cancer type. Second, we obtained omics features for each gene using gene expression and gene mutation data. After that, we implemented the same experiment by utilizing a procedure similar to stratified 10-fold CV. The datasets used include ten disjoint sets of negative samples (i.e., non-target genes) and one set of positive samples (i.e., target genes) for each cancer type. Thus, we have in total 390 (bladder), 870 (breast), 830 (colon), 320 (kidney), 2280 (leukemia), 270 (liver), 670 (lung) non-target genes per cancer type. For example, when we have 39 target genes for bladder cancer, we used 390 non-target genes (divided into 10 sets of 39), which means we have ten times more non-target genes than target genes for each cancer type. Each of the ten negative sample (non-target gene) sets was separately combined with the same positive sample, shuffled, and then randomly split into training and test sets (70% for training and 30% for testing) in a stratified fashion to preserve each class label distribution. We report results in terms of AUC in the test set. We repeated this process ten times and averaged the results across all test sets. Finally, we compared the prediction performance (in terms of AUC) of the best OncoRTT model with the best model for the previous work (Bazaga et al., 2020) in seven cancer types, common to our work and the previous work (See Figure 4). OncoRTT outperformed the baseline method by 11%, 11%, 11%, 20%, and 2% in the bladder, breast, colon, leukemia, and liver cancers, respectively. On the other hand, OncoRTT achieved an AUC lower by 7% and 3% in kidney and lung cancers, respectively. However, OncoRTT obtained an average AUC better than the baseline method by 6.5% across all seven cancers. Despite using the other methods’ leukemia dataset, OncoRTT again achieved the best performance for the leukemia dataset.

We believe that this result is a consequence of the leukemia dataset once again having a larger number of positive and negative samples and DL being more effective when dealing with more extensive data. Moreover, the result suggests that the main advantages/features that make OncoRTT more efficient and powerful are.

• In our study, we utilized the state-of-the-art method that generates protein sequence embeddings called ProtTrans BERT-based embedding, but in the baseline method, they utilized PPI embeddings. ProtTrans trained two autoregressive models (Transformer-X and XLNet) and four auto-encoder models (BERT, Albert, Electra, and T5) using more than two thousand million protein sequences, making it the state-of-the-art method to encode the protein sequence with the highest quality embeddings. ProtTrans-BERT captures different biophysical properties of the amino acids, structure classes of proteins, domains of life and viruses, and protein functions in conserved motifs.
• Using features from the amino-acid sequences strengthens our method because amino-acid sequences are available for any protein, while the protein interaction profile is unavailable for many proteins.
• Similar to the baseline method, the second source of our features is omics data. However, instead of using the average of gene expression values over the patients, we aggregate the GE values using four different functions, giving a better and broader representation of the GE features and thus enhancing the prediction performance.
• We used a DNN classifier for prediction, which can learn arbitrary boundaries and thus handle the binary classification better than decision trees (i.e., RF, the baseline classifier). Since we have a high number of features, the DNN works better than ML classifiers.

Beyond that, we also provide the comprehensive datasets, OncologyTT, which is richer than the baseline datasets. That is, we increased the number of targets by collecting from two reliable sources (DrugBank and the TTD database).

It is important to mention that in our datasets, OncologyTT, the negative samples in each fold of the 10-fold CV were the same negative genes, while in the baseline datasets, the negative samples are different in each fold with no overlap. Our method, OncoRTT, utilized supervised learning classifiers (i.e., took the features and their labels as input) and performed well by achieving high AUC using OncologyTT and the baseline datasets. This result demonstrated the capability of our method and the crucial distinct features that we extracted for each gene that helps the classifier differentiate between negative and positive samples (i.e., target and non-target genes). Moreover, when we evaluated the prediction performance, we calculated the AUC for each fold and then averaged the results over the 10-fold CV for both experiments, which mitigates possible bias and minimizes the variance in the classification performance.

### 4.3 Novel therapeutic cancer targets prediction and validation

To further demonstrate OncoRTT’s use, we conducted several new experiments to predict new therapeutic targets for each cancer type separately. To perform these experiments, we utilized two datasets. The first is the OncologyTT dataset used to build and train our model, and the second is the Cancer-Target baseline dataset used as independent unseen test data. Firstly, we considered all the negative genes in this test data as unlabeled genes collected from a pool of human protein-coding genes. Thus, we focused on these unlabeled genes to predict the potential novel target genes. We implemented the following procedure per cancer type: first, we retrained the best model (i.e., the DNN classifier with integrated FVs) using the whole OncologyTT dataset. Second, we used this model for prediction using the unseen test data. Third, to utilize the prediction results, we increased the number of epochs to 100 since the data samples are much higher. In this manner, all the negative genes in the test data predicted to be positive (i.e., target genes) are collected and ranked based on their prediction probability scores. We further analyzed the results of the top-10 ranked targets related to three cancer types: colon, kidney, and lung cancers, listed in Tables 6; Tables 7; Tables 8, respectively. Supplementary Tables S3–S6 provide the top-10 ranked targets for the other cancers (bladder, breast, liver, and leukemia).

**TABLE 6:** Top 10-ranked novel predicted therapeutic targets based on the prediction score for **colon** cancer with the number and type of evidence linking the targets and disease.

Gene	UniProt ID	Prediction score	Protein name	# Of evidence	Validation evidence type
EEF1A1	P68104	0.9969	Eukaryotic Translation Elongation Factor 1 Alpha	8	Text mining
HP	P00738	0.9892	Haptoglobin	6	Text mining, RNA expression
CHL1	O00533	0.9886	Cell Adhesion Molecule L1 Like	6	Text mining, RNA expression
ADIPOQ	Q15848	0.9866	Adiponectin, C1Q And Collagen Domain Containing	74	Text mining, RNA expression
MYLK4	Q86YV6	0.9857	Myosin Light Chain Kinase Family Member 4	0	Linked to one cancer
IGFBP5	P24593	0.9801	Insulin Like Growth Factor Binding Protein 5	5	Text mining, RNA expression
ILK	Q13418	0.9785	Integrin Linked Kinase	13	Text mining, Pathways
TFF1	P04155	0.9756	Trefoil Factor 1	5	Text mining, RNA expression, Genetic association
EPHB2	P29323	0.9727	EPH Receptor B2	42	Text mining, RNA expression, Somatic mutations, Drugs
EPHA7	Q15375	0.9722	EPH Receptor A7	18	Text mining, RNA expression, Somatic mutations, Drugs

**TABLE 7:** Top 10-ranked novel predicted therapeutic targets based on the prediction score for **kidney** cancer with the number and type of evidence linking the targets and disease.

Gene	UniProt ID	Prediction score	Protein name	# Of evidence	Association type
LY96	Q9Y6Y9	0.9778	Lymphocyte Antigen 96	1	RNA Expression
WNK2	Q9Y3S1	0.9778	WNK Lysine Deficient Protein Kinase 2	30	Text mining, Somatic mutation
FMO3	P31513	0.9777	Flavin Containing Dimethylaniline Monoxygenase 3	1	Renal cell carcinoma
					RNA Expression
PRKCB	P05771	0.9777	Protein Kinase C Beta	6	Somatic mutations, RNA expression, and Drugs
CDCA8	Q53HL2	0.9774	Cell Division Cycle Associated 8	6	Text mining, RNA expression
					Renal cell carcinoma
HSPB6	O14558	0.9773	Heat Shock Protein Family B (Small) Member 6	0	Linked to acute kidney disease
FOSL1	P15407	0.9772	FOS Like 1, AP-1 Transcription Factor Subunit	4	Renal cell carcinoma
					Text Mining
TRIM55	Q9BYV6	0.9771	Tripartite Motif Containing 55	0	Linked to **colorectal cancer**
FBLN7	Q53RD9	0.9769	Fibulin 7	0	Linked to other **kidney** diseases
RARB	P10826	0.9760	Retinoic Acid Receptor Beta	13	Text mining, Drugs

**TABLE 8:** Top 10-ranked novel predicted therapeutic targets based on the prediction score for **lung** cancer with the number and type of evidence linking the targets and disease.

Gene	UniProt ID	Prediction score	Protein name	# Of evidence	Type of associations
ACMSD	Q8TDX5	0.9857	Aminocarboxymuconate Semialdehyde Decarboxylase	0	Linked to other **cancers**
ONECUT2	O95948	0.9615	One Cut Homeobox 2	8	Text mining, RNA expression
RPL10L	Q96L21	0.9568	Ribosomal Protein L10 Like	0	Linked to other **cancer**
HIST1H2BL (H2BC13)	Q99880	0.9363	H2B Clustered Histone 13	5	Text mining, Genetic association, RNA expression
ALDH8A1	Q9H2A2	0.9322	Aldehyde Dehydrogenase 8 Family Member A1	0	Linked to other **cancer**
CACNA1S	Q13698	0.9199	Calcium Voltage-Gated Channel Subunit Alpha1 S	6	Text mining, RNA expression, Pathways
CAV3	P56539	0.9151	Caveolin 3	1	RNA expression
CNTN5	O94779	0.8685	Contactin 5	1	Text mining
MAATS1	Q7Z4T9	0.8396	MYCBP/AMY-1-Associated Testis-Expressed Protein 1	1	RNA expression
EPHA5	P54756	0.8097	EPH Receptor A5	33	Text mining, Somatic mutation, Pathways, Drugs

We used the Open Targets Platform to verify each cancer’s novel predicted therapeutic targets (Koscielny et al., 2017). This comprehensive tool supports the systematic identification and prioritization of potential therapeutic targets. In addition, it provides potentially causal evidence linking targets and diseases, which we applied to link the top predicted targets to specific cancer types using six of the association methods offered by this platform, which are.

1. **Text mining:** Evaluating the strength of the association between the target gene and specific cancer type using their co-occurrence in the published literature.
2. **Genetic associations:** Phenome-wide associated loci prioritizing the target genes as a likely causal gene for specific cancers.
3. **Somatic mutation:** A catalogue of somatic mutations that causally implicate the target genes in specific cancers.
4. **Drugs:** Clinical candidates and (or) approved drugs pharmacologically targeting the target genes and indicated for the specific cancers-the source of approved interaction in the ChEMBL database.
5. **Pathways and system biology:** Multiple pathway analysis tools providing enrichment based on genomic alterations associating the target gene with specific cancers.
6. **RNA expression**: Transcriptomic analysis reports a significant differential expression of the target gene when comparing control samples with specific cancer samples.

Tables 6, Tables 7, and Tables 8 lists the top-10 ranked novel target genes for colon, kidney, and lung cancer, respectively, with their prediction probability scores, the number, and the type of validation evidence. When we did not find any association to the specific cancer type, we put ‘0'. However, we specify if this gene is linked to other cancers or the organ associated with the cancer progression.

For the top predicted genes in colon cancer, we verified 90% of the top predicted genes by finding different types of evidence (see Table 6). The results indicate that the most crucial gene is *ADIPOQ* ***,*** linked to colon, colorectal, and metastasis colorectal cancers (Yang et al., 2015; Deng et al., 2020). The possible reason that *ADIPOQ* may play a role in cell growth, angiogenesis, and tissue remodeling is by binding and sequestering various growth factors with distinct binding affinities (Sakellariou et al., 2016). All genes were associated with colon cancer based on the Open Targets Platform, except one, *MYLK4*. However, *MYLK4* is associated with squamous cell carcinoma. Furthermore, based on Expression Atlas, transcriptomic and RNA-seq analysis tools show seven of the ten targets were significantly differentially expressed in colon cancer (Papatheodorou et al., 2020).

For kidney cancer (see Table 7), the Open Targets Platform links seven of the top predicted targets to kidney cancer. Specifically, four are linked to kidney cancer, while the three other genes (*CDCA8, FOSL1,* and *FMO3*) are linked explicitly to renal cell carcinoma, an aggressive kidney cancer originating in the lining of the proximal convoluted tubule (a part of the tiny tubes in the kidney) that primarily transport urine (Cohen and McGovern, 2005). *WNK2* obtained the highest evidence, a critical kinase gene that has a crucial role in regulating electrolyte homeostasis, cell signaling, survival, and proliferation. In addition, the catalogue of somatic mutations that causally implicate *WNK2* in kidney neoplasm, and several published studies connect this gene to different kidney cancers. Interestingly, although we did not find any connection for *TRIM55, HSPB6, and FBLN7* to kidney cancer, all these genes are associated with several other kidney diseases, indicating model capability to find the hidden patterns connecting the genes to specific organs of interest (Bleyer et al., 2017; Ullah et al., 2020).

For lung cancer (see Table 8), we found evidence linking seven predicted targets to the disease. Even though there is no evidence associating the first predicted gene, ACMSD, with lung cancer, this gene is linked to colorectal, pancreatic ductal, chromophobe renal cell, breast, and brain cancers, indicating its essential role. *CAV3*, *CNTN5*, and *MAATS1* have only one line of evidence linking them to lung cancer, but the Open Targets Platform links them to other cancers in the primary or metastasis stages. The 10th predicted therapeutic target, *EPHA5,* has the most significant number of evidence linking it to lung cancer. The top-10 predicted therapeutic targets for lung cancer are further discussed in the case study below.

To summarize, Tables 6, 7, and 8 (and Supplementary Tables S3–S6) have several lines of evidence linking the predicted therapeutic targets to the specific cancer type. These results increase confidence in the power of our approach for predicting therapeutic targets, which experimental researchers can further explore for anticancer drug development and repositioning.

### 4.4 Lung cancer case study: Findings that support the predicted novel targets

A comprehensive understanding of specific cancer types and the hallmarks of each are essential for effective cancer treatment. In cancer treatment, physicians use drugs to target specific genes (or proteins) related to the tumor-cell growth and survival (Chatterjee and Bivona, 2019). Therefore, we further explored the top-10 predicted therapeutic targets for lung cancer by performing differential expression analysis (DEA) to identify if the top-10 predicted therapeutic targets are DEGs, using the TCGAbiolinks package implemented in R (Colaprico et al., 2016).

First, we accessed “TCGA-LUAD” and “TCGA-LUSC” to collect around 58 TCGA tumor samples for lung cancer patients and 58 corresponding normal TCGA samples and obtained their relevant transcriptome profiling and gene expression quantification data. Then, we compared the normal and primary tumor samples using pair-wise tests to obtain the differential expression genes between these two groups. Finally, we filtered the DEGs output by determining a cutoff threshold from the *p*-values <0.05. Table 9 shows that eight of the top-10 predicted therapeutic targets are DEGs. *p*-values and the adjusted *p*-values (FDR) in Table 9 show that eight genes are significantly expressed among the top-10 predicted genes, obtaining *p*-values <0.05, which provides experimental support for our predicted therapeutic targets.

**TABLE 9:** The top-10 predicted therapeutic targets for lung cancer identified as DEGs and ranked based on their *p*-value. We also provide: FDR, an adjusted *p*-value, as a correction of the expression level; LogCPM (the log count per million), a measure of expression level; and logFC (the log fold-change), which is the log difference between the normal and primary tumor groups.

Gene	*p*-value	LogFC	LogCPM	FDR
ONECUT2	2.89E-35	−3.9579868	0.152915076	7.76E-34
CAV3	8.08E-34	3.84724947	−1.054627696	1.94E-32
ACMSD	2.31E-17	−3.2579084	0.10877721	1.46E-16
HIST1H2BL	5.65E-13	−4.1565072	−1.807926281	2.44E-12
CACNA1S	2.15E-11	3.09338945	−0.197572093	8.03E-11
MAATS1	7.89E-05	0.70988044	3.15065947	0.000207677
CNTN5	0.000911647	−0.6029651	0.671936123	0.002054721
ALDH8A1	0.001792398	0.577664	−0.428207734	0.00385545
EPHA5	N/A	N/A	N/A	N/A
RPL10L	N/A	N/A	N/A	N/A

In our analysis, *EPHA5* and *RPL10L* were not classified as DEGs based on the normal and primary tumor samples using the cutoff mentioned above. There is no evidence linking *RPL10L* to lung cancer, although it is linked to other cancer types. However, *EPHA5* is associated with lung cancer. We found clinical candidates, and an approved drug (VANDETANIB) targeting *EPHA5* has been indicated for non-small cell lung carcinoma at different phases. However, these studies’ status is currently only defined as either “completed” or “active, not recruiting” (Ochoa et al., 2021). Beyond that, the most significantly expressed gene, *ONECUT2*, is associated with five lung cancer categories including lung adenocarcinoma, non-small cell lung carcinoma, small cell lung carcinoma, lung carcinoma, and lung carcinoid tumor. On the other hand, the third significantly expressed gene, *ACMSD*, has not been linked to lung cancer. However, ACMSD ultimately controls the metabolic fate of tryptophan catabolism along the Kynurenine pathway. This is interesting as Tryptophan is converted to Kynurenine, and the Kynurenine/Tryptophan Ratio has recently been reported as a potential blood-based biomarker in non-small cell lung cancer (Mandarano et al., 2021). Also, the modulation of Tryptophan metabolism has been used for diagnosis, prognosis, and therapies in lung cancer (Li and Zhao, 2021), and the Kynurenine pathway is being targeted for the treatment of Cisplatin-resistant lung cancer through inhibiting or knocking down indoleamine 2,3-dioxygenase-1 (*IDO1*) (Nguyen et al., 2020). However, targeting the Kynurenine pathway *via ACMSD* from the same enzyme-inhibitory activity and antitumor efficacy standpoint has not been accessed.

Beyond this, we performed a MirDB search (http://www.mirdb.org/), an online database to predict functional microRNA targets (Chen and Wang, 2020), in November 2021. We found ONECUT2, MAATS1, CNTN5, and EPHA5 predicted to be controlled by the same microRNA, hsa-miR-1267. We also found ONECUT2, MAATS1, and EPHA5 predicted to be controlled by another microRNA, hsa-miR-203a-3p, as well. Both hsa-miR-1267 and hsa-miR-203a-3p are two of a 24 panel of circulating microRNA in plasma, reported by Wozniak and colleagues (Wozniak et al., 2015), capable of discriminating lung cancer cases from non-cancer controls (AUC of 0.92). DEGs are vital to understanding the biological differences between healthy and diseased states, and hence they can be useful to pinpoint candidate therapeutic targets or gene signatures for diagnostics (Rodriguez-Esteban and Jiang, 2017).

## 5 Conclusion

Combining AI and ML/DL with pharmacology made the development of several applications to solve diverse biomedical domain problems possible. Here, we attempted to use the same strategy to create a solution for oncology-related therapeutic target identification, which is currently the main challenge for anticancer drug development and repurposing. We developed OncoRTT that exploited the power of the BERT technique and DL to identify therapeutic targets efficiently. Specifically, we auto-generated feature representations (i.e., embeddings) by applying BERT to the proteins’ amino-acid sequence per cancer type. We also extracted omics features using gene expression and gene mutation data. Finally, we combined these features and fed them to the DNN models for prediction. We additionally created datasets, OncologyTT, to build, train, and test our model. OncoRTT demonstrated its ability to differentiate between cancer-specific type target genes and non-target genes by achieving high AUC. Furthermore, OncoRTT achieved better prediction performance than the baseline method in most cancer types and, on average, across all the cancer types common to both studies. The obtained results indicate that the performance of DL classifiers exceeded the ML classifiers in most cases despite the DL model’s capabilities being limited by the small number of positive targets and more data needed to build and train DL models. The last limitation to highlight is the lack of interpretability of the BERT embeddings (i.e., feature representation vector), preventing gaining insight into the critical features. Thus, as a future direction, the output of DL-based models should be made more interpretable and meaningful for bioinformaticians and experimental scientists.

For further improvements to predict novel therapeutic targets, we suggest.

- Applying other embedding techniques such as graph convolutional neural network (GCN) on PPI to generate latent feature representation of each gene.
- Integrating more omics features such as copy number variants.
- Utilizing different ML/DL classifiers.
- Making the DL models more interpretable in terms of feature extraction and classification.

Also, we plan to extend our work in several directions, including.

- Upgrade the OncologyTT dataset by including more data samples for the current cancers and incorporating more cancers
- Validating the novel predicted therapeutic targets.
- Our ongoing project aims to predict oncology-related DTIs for the newly identified targets provided by our method, OncoRTT, and for the existing targets using our DTi2Vec tools (Thafar et al., 2021). In addition, this process will allow us to predict new anticancer drugs that will subsequently be tested by predicting the drugs’ response in cancer cell lines.

To our knowledge, this is among the few studies to consolidate data from several resources per cancer type and then identify novel therapeutic targets per cancer using an ML/DL approach. Beyond that, our findings pinpoint some essential proteins per cancer type that could be possible therapeutic targets, for which we found several lines of evidence linking them to the specific cancer types. Non-etheless, follow-up experiments should be performed to validate these novel therapeutic targets.

## Data Availability

The datasets presented in this study can be found in online repositories. The names of the repository/repositories and accession number(s) can be found in the article/Supplementary Material.
