# Supplementary material for: OncoRTT: Predicting novel oncology-related therapeutic targets using BERT embeddings and omics features
Source: Front Genet. 2023 Apr 6;14:1139626. doi: 10.3389/fgene.2023.1139626 (PMC10117673; doi:10.3389/fgene.2023.1139626)
Supplement: Supplementary file 1 [file Table1.docx]

**OncoRTT: Predicting Novel Oncology-related Therapeutic Targets using BERT Embeddings and Omics Features**

Maha A. Thafar ^1,2^, Somayah Albaradei^1,3^, Mahmut Uludag^1^, Mona Alshahrani^4^, Takashi Gojobori^1^, Magbubah Essack^1*^, Xin Gao^1*^

^1^ Computer, Electrical and Mathematical Sciences and Engineering Division (CEMSE), Computational Bioscience Research Center, Computer (CBRC), King Abdullah University of Science and Technology (KAUST), Thuwal, Kingdom of Saudi Arabia.

^2^ College of Computers and Information Technology, Computer Science Department, Taif University, Taif, Kingdom of Saudi Arabia.

^3^ Faculty of Computing and Information Technology, King Abdulaziz University, Jeddah, Kingdom of Saudi Arabia.

^4^ National Center for Artificial Intelligence (NCAI), Saudi Data and Artificial Intelligence Authority (SDAIA), Riyadh, Saudi Arabia

**Supplementary Section 1: The details of collecting the biomarker cancer genes.**

From the Catalogue of Somatic Mutations in Cancer (COSMIC) database that provides all gene expression level 3 data from the cancer genome atlas (TCGA) database, we downloaded the Gene expression data (GE). From the GE data, we used the following attributes:

- *Sample id:* a sample is a portion of a tumor being examined. A number of samples can be taken from a single tumor and a number of tumors can be obtained from one individual. A *sample id* is used to identify a sample within the COSMIC database. The samples are from ICGC and TCGA. However, we excluded the ICGA cases.
- *Gene name*: the accepted HGNC identifier.
- *Regulation:* the over or under expressed status depending on the scores from different platforms if they are above or below the threshold.
- *Z-score:* an indicative score of gene expression taken from different platforms.

We used the regulation attribute to filter the GE by getting just the significantly over-regulated genes. However, the GE data did not link the over-regulated genes to any cancer type. Therefore, we connected all over-regulated genes to specific cancer types using the metadata from the Genomic Data Commons Data Portal (GDC) <https://portal.gdc.cancer.gov/> [34]. To do this, for each cancer type in our list, we made a query by filtering the cases using 'TCGA samples', 'primary tumor', and specifying the disease type, and then downloaded 10 for each cancer type (see Supplementary Table S1). Then, to find the over-regulated gene associated with each cancer type, we matched the sample IDs for all the over-regulated genes from the GE data with the TCGA sample IDs downloaded from the GDC. After obtaining a list of over-regulated genes for each cancer type, we sorted them using the z-scores and added the top 100 genes to the target gene data for each cancer.

### **Supplementary Table S1:** The number of TCGA samples and their properties that download using GDC Portal to obtain the over-regulated genes as positive genes

| **Cancer Type** | **GDC Project-ID** | **GDC Study Name** | **Sample type** | **Tissue type** |
| --- | --- | --- | --- | --- |
| ***Bladder*** | [TCGA-BLCA](https://portal.gdc.cancer.gov/exploration?filters=%7B%22op%22%3A%22and%22%2C%22content%22%3A%5B%7B%22content%22%3A%7B%22field%22%3A%22cases.diagnoses.tissue_or_organ_of_origin%22%2C%22value%22%3A%5B%22anterior%20wall%20of%20bladder%22%2C%22bladder%20neck%22%2C%22bladder%2C%20nos%22%2C%22dome%20of%20bladder%22%2C%22lateral%20wall%20of%20bladder%22%2C%22overlapping%20lesion%20of%20bladder%22%2C%22posterior%20wall%20of%20bladder%22%2C%22trigone%20of%20bladder%22%2C%22urachus%22%2C%22ureteric%20orifice%22%5D%7D%2C%22op%22%3A%22in%22%7D%2C%7B%22content%22%3A%7B%22field%22%3A%22cases.primary_site%22%2C%22value%22%3A%5B%22bladder%22%5D%7D%2C%22op%22%3A%22in%22%7D%2C%7B%22op%22%3A%22in%22%2C%22content%22%3A%7B%22field%22%3A%22cases.project.program.name%22%2C%22value%22%3A%5B%22TCGA%22%5D%7D%7D%2C%7B%22op%22%3A%22in%22%2C%22content%22%3A%7B%22field%22%3A%22cases.project.project_id%22%2C%22value%22%3A%5B%22TCGA-BLCA%22%5D%7D%7D%5D%7D) | Bladder Urothelial Carcinoma | Primary tumor | [transitional cell papillomas and carcinomas](https://portal.gdc.cancer.gov/exploration?filters=%7B%22op%22%3A%22and%22%2C%22content%22%3A%5B%7B%22content%22%3A%7B%22field%22%3A%22cases.diagnoses.tissue_or_organ_of_origin%22%2C%22value%22%3A%5B%22anterior%20wall%20of%20bladder%22%2C%22bladder%20neck%22%2C%22bladder%2C%20nos%22%2C%22dome%20of%20bladder%22%2C%22lateral%20wall%20of%20bladder%22%2C%22overlapping%20lesion%20of%20bladder%22%2C%22posterior%20wall%20of%20bladder%22%2C%22trigone%20of%20bladder%22%2C%22urachus%22%2C%22ureteric%20orifice%22%5D%7D%2C%22op%22%3A%22in%22%7D%2C%7B%22op%22%3A%22in%22%2C%22content%22%3A%7B%22field%22%3A%22cases.disease_type%22%2C%22value%22%3A%5B%22transitional%20cell%20papillomas%20and%20carcinomas%22%5D%7D%7D%2C%7B%22content%22%3A%7B%22field%22%3A%22cases.primary_site%22%2C%22value%22%3A%5B%22bladder%22%5D%7D%2C%22op%22%3A%22in%22%7D%2C%7B%22op%22%3A%22in%22%2C%22content%22%3A%7B%22field%22%3A%22cases.project.program.name%22%2C%22value%22%3A%5B%22TCGA%22%5D%7D%7D%2C%7B%22op%22%3A%22in%22%2C%22content%22%3A%7B%22field%22%3A%22cases.samples.sample_type%22%2C%22value%22%3A%5B%22primary%20tumor%22%5D%7D%7D%5D%7D) |
| ***Breast*** | [TCGA-BRCA](https://portal.gdc.cancer.gov/exploration?filters=%7B%22op%22%3A%22and%22%2C%22content%22%3A%5B%7B%22content%22%3A%7B%22field%22%3A%22cases.diagnoses.tissue_or_organ_of_origin%22%2C%22value%22%3A%5B%22axillary%20tail%20of%20breast%22%2C%22breast%2C%20nos%22%2C%22central%20portion%20of%20breast%22%2C%22lower-inner%20quadrant%20of%20breast%22%2C%22lower-outer%20quadrant%20of%20breast%22%2C%22nipple%22%2C%22overlapping%20lesion%20of%20breast%22%2C%22upper-inner%20quadrant%20of%20breast%22%2C%22upper-outer%20quadrant%20of%20breast%22%5D%7D%2C%22op%22%3A%22in%22%7D%2C%7B%22content%22%3A%7B%22field%22%3A%22cases.primary_site%22%2C%22value%22%3A%5B%22breast%22%5D%7D%2C%22op%22%3A%22in%22%7D%2C%7B%22op%22%3A%22in%22%2C%22content%22%3A%7B%22field%22%3A%22cases.project.program.name%22%2C%22value%22%3A%5B%22TCGA%22%5D%7D%7D%2C%7B%22op%22%3A%22in%22%2C%22content%22%3A%7B%22field%22%3A%22cases.project.project_id%22%2C%22value%22%3A%5B%22TCGA-BRCA%22%5D%7D%7D%5D%7D) | Breast invasive carcinoma | Primary tumor | [complex epithelial neoplasms](https://portal.gdc.cancer.gov/exploration?filters=%7B%22op%22%3A%22and%22%2C%22content%22%3A%5B%7B%22content%22%3A%7B%22field%22%3A%22cases.diagnoses.tissue_or_organ_of_origin%22%2C%22value%22%3A%5B%22axillary%20tail%20of%20breast%22%2C%22breast%2C%20nos%22%2C%22central%20portion%20of%20breast%22%2C%22lower-inner%20quadrant%20of%20breast%22%2C%22lower-outer%20quadrant%20of%20breast%22%2C%22nipple%22%2C%22overlapping%20lesion%20of%20breast%22%2C%22upper-inner%20quadrant%20of%20breast%22%2C%22upper-outer%20quadrant%20of%20breast%22%5D%7D%2C%22op%22%3A%22in%22%7D%2C%7B%22content%22%3A%7B%22field%22%3A%22cases.primary_site%22%2C%22value%22%3A%5B%22breast%22%5D%7D%2C%22op%22%3A%22in%22%7D%2C%7B%22op%22%3A%22in%22%2C%22content%22%3A%7B%22field%22%3A%22cases.project.program.name%22%2C%22value%22%3A%5B%22TCGA%22%5D%7D%7D%2C%7B%22op%22%3A%22in%22%2C%22content%22%3A%7B%22field%22%3A%22cases.samples.sample_type%22%2C%22value%22%3A%5B%22primary%20tumor%22%5D%7D%7D%5D%7D) |
| ***Colon*** | [TCGA-COAD](https://portal.gdc.cancer.gov/exploration?filters=%7B%22op%22%3A%22and%22%2C%22content%22%3A%5B%7B%22content%22%3A%7B%22field%22%3A%22cases.diagnoses.tissue_or_organ_of_origin%22%2C%22value%22%3A%5B%22appendix%22%2C%22ascending%20colon%22%2C%22cecum%22%2C%22colon%2C%20nos%22%2C%22descending%20colon%22%2C%22hepatic%20flexure%20of%20colon%22%2C%22overlapping%20lesion%20of%20colon%22%2C%22rectosigmoid%20junction%22%2C%22rectum%2C%20nos%22%2C%22sigmoid%20colon%22%2C%22splenic%20flexure%20of%20colon%22%2C%22transverse%20colon%22%5D%7D%2C%22op%22%3A%22in%22%7D%2C%7B%22content%22%3A%7B%22field%22%3A%22cases.primary_site%22%2C%22value%22%3A%5B%22colon%22%2C%22rectosigmoid%20junction%22%2C%22rectum%22%5D%7D%2C%22op%22%3A%22in%22%7D%2C%7B%22op%22%3A%22in%22%2C%22content%22%3A%7B%22field%22%3A%22cases.project.program.name%22%2C%22value%22%3A%5B%22TCGA%22%5D%7D%7D%2C%7B%22op%22%3A%22in%22%2C%22content%22%3A%7B%22field%22%3A%22cases.project.project_id%22%2C%22value%22%3A%5B%22TCGA-COAD%22%5D%7D%7D%5D%7D) | Colon adenocarcinoma | Primary tumor | [cystic, mucinous, and serous neoplasms](https://portal.gdc.cancer.gov/exploration?filters=%7B%22op%22%3A%22and%22%2C%22content%22%3A%5B%7B%22content%22%3A%7B%22field%22%3A%22cases.diagnoses.tissue_or_organ_of_origin%22%2C%22value%22%3A%5B%22appendix%22%2C%22ascending%20colon%22%2C%22cecum%22%2C%22colon%2C%20nos%22%2C%22descending%20colon%22%2C%22hepatic%20flexure%20of%20colon%22%2C%22overlapping%20lesion%20of%20colon%22%2C%22rectosigmoid%20junction%22%2C%22rectum%2C%20nos%22%2C%22sigmoid%20colon%22%2C%22splenic%20flexure%20of%20colon%22%2C%22transverse%20colon%22%5D%7D%2C%22op%22%3A%22in%22%7D%2C%7B%22content%22%3A%7B%22field%22%3A%22cases.primary_site%22%2C%22value%22%3A%5B%22colon%22%2C%22rectosigmoid%20junction%22%2C%22rectum%22%5D%7D%2C%22op%22%3A%22in%22%7D%2C%7B%22op%22%3A%22in%22%2C%22content%22%3A%7B%22field%22%3A%22cases.project.program.name%22%2C%22value%22%3A%5B%22TCGA%22%5D%7D%7D%5D%7D) |
| ***Kidney*** | [TCGA-KIRC](https://portal.gdc.cancer.gov/exploration?filters=%7B%22op%22%3A%22and%22%2C%22content%22%3A%5B%7B%22content%22%3A%7B%22field%22%3A%22cases.diagnoses.tissue_or_organ_of_origin%22%2C%22value%22%3A%5B%22kidney%2C%20nos%22%5D%7D%2C%22op%22%3A%22in%22%7D%2C%7B%22content%22%3A%7B%22field%22%3A%22cases.primary_site%22%2C%22value%22%3A%5B%22kidney%22%5D%7D%2C%22op%22%3A%22in%22%7D%2C%7B%22op%22%3A%22in%22%2C%22content%22%3A%7B%22field%22%3A%22cases.project.program.name%22%2C%22value%22%3A%5B%22TCGA%22%5D%7D%7D%2C%7B%22op%22%3A%22in%22%2C%22content%22%3A%7B%22field%22%3A%22cases.project.project_id%22%2C%22value%22%3A%5B%22TCGA-KIRC%22%5D%7D%7D%5D%7D)  TCGA-KICH | Kidney renal clear cell carcinoma, and kidney renal papillary cell carcinoma | Primary tumor | [adenomas and adenocarcinomas](https://portal.gdc.cancer.gov/exploration?filters=%7B%22op%22%3A%22and%22%2C%22content%22%3A%5B%7B%22content%22%3A%7B%22field%22%3A%22cases.diagnoses.tissue_or_organ_of_origin%22%2C%22value%22%3A%5B%22kidney%2C%20nos%22%5D%7D%2C%22op%22%3A%22in%22%7D%2C%7B%22op%22%3A%22in%22%2C%22content%22%3A%7B%22field%22%3A%22cases.disease_type%22%2C%22value%22%3A%5B%22adenomas%20and%20adenocarcinomas%22%5D%7D%7D%2C%7B%22content%22%3A%7B%22field%22%3A%22cases.primary_site%22%2C%22value%22%3A%5B%22kidney%22%5D%7D%2C%22op%22%3A%22in%22%7D%2C%7B%22op%22%3A%22in%22%2C%22content%22%3A%7B%22field%22%3A%22cases.project.program.name%22%2C%22value%22%3A%5B%22TCGA%22%5D%7D%7D%2C%7B%22op%22%3A%22in%22%2C%22content%22%3A%7B%22field%22%3A%22cases.samples.sample_type%22%2C%22value%22%3A%5B%22primary%20tumor%22%5D%7D%7D%5D%7D) |
| ***Leukemia*** | [TCGA-LAML](https://portal.gdc.cancer.gov/exploration?filters=%7B%22op%22%3A%22and%22%2C%22content%22%3A%5B%7B%22content%22%3A%7B%22field%22%3A%22cases.diagnoses.tissue_or_organ_of_origin%22%2C%22value%22%3A%5B%22blood%22%2C%22bone%20marrow%22%2C%22hematopoietic%20system%2C%20nos%22%2C%22reticuloendothelial%20system%2C%20nos%22%2C%22spleen%22%5D%7D%2C%22op%22%3A%22in%22%7D%2C%7B%22op%22%3A%22in%22%2C%22content%22%3A%7B%22field%22%3A%22cases.disease_type%22%2C%22value%22%3A%5B%22myeloid%20leukemias%22%5D%7D%7D%2C%7B%22content%22%3A%7B%22field%22%3A%22cases.primary_site%22%2C%22value%22%3A%5B%22hematopoietic%20and%20reticuloendothelial%20systems%22%5D%7D%2C%22op%22%3A%22in%22%7D%5D%7D) | Acute Myeloid Leukemia | [Primary blood derived cancer, and peripheral blood](https://portal.gdc.cancer.gov/exploration?filters=%7B%22op%22%3A%22and%22%2C%22content%22%3A%5B%7B%22content%22%3A%7B%22field%22%3A%22cases.diagnoses.tissue_or_organ_of_origin%22%2C%22value%22%3A%5B%22blood%22%2C%22bone%20marrow%22%2C%22hematopoietic%20system%2C%20nos%22%2C%22reticuloendothelial%20system%2C%20nos%22%2C%22spleen%22%5D%7D%2C%22op%22%3A%22in%22%7D%2C%7B%22op%22%3A%22in%22%2C%22content%22%3A%7B%22field%22%3A%22cases.disease_type%22%2C%22value%22%3A%5B%22myeloid%20leukemias%22%5D%7D%7D%2C%7B%22content%22%3A%7B%22field%22%3A%22cases.primary_site%22%2C%22value%22%3A%5B%22hematopoietic%20and%20reticuloendothelial%20systems%22%5D%7D%2C%22op%22%3A%22in%22%7D%2C%7B%22op%22%3A%22in%22%2C%22content%22%3A%7B%22field%22%3A%22cases.project.program.name%22%2C%22value%22%3A%5B%22TCGA%22%5D%7D%7D%5D%7D) | [myeloid leukemia](https://portal.gdc.cancer.gov/exploration?filters=%7B%22op%22%3A%22and%22%2C%22content%22%3A%5B%7B%22content%22%3A%7B%22field%22%3A%22cases.diagnoses.tissue_or_organ_of_origin%22%2C%22value%22%3A%5B%22blood%22%2C%22bone%20marrow%22%2C%22hematopoietic%20system%2C%20nos%22%2C%22reticuloendothelial%20system%2C%20nos%22%2C%22spleen%22%5D%7D%2C%22op%22%3A%22in%22%7D%2C%7B%22content%22%3A%7B%22field%22%3A%22cases.primary_site%22%2C%22value%22%3A%5B%22hematopoietic%20and%20reticuloendothelial%20systems%22%5D%7D%2C%22op%22%3A%22in%22%7D%2C%7B%22op%22%3A%22in%22%2C%22content%22%3A%7B%22field%22%3A%22cases.project.program.name%22%2C%22value%22%3A%5B%22TCGA%22%5D%7D%7D%2C%7B%22op%22%3A%22in%22%2C%22content%22%3A%7B%22field%22%3A%22cases.samples.sample_type%22%2C%22value%22%3A%5B%22primary%20blood%20derived%20cancer%20-%20peripheral%20blood%22%5D%7D%7D%5D%7D) |
| ***Liver*** | [TCGA-LIHC](https://portal.gdc.cancer.gov/exploration?filters=%7B%22op%22%3A%22and%22%2C%22content%22%3A%5B%7B%22content%22%3A%7B%22field%22%3A%22cases.diagnoses.tissue_or_organ_of_origin%22%2C%22value%22%3A%5B%22intrahepatic%20bile%20duct%22%2C%22liver%22%5D%7D%2C%22op%22%3A%22in%22%7D%2C%7B%22content%22%3A%7B%22field%22%3A%22cases.primary_site%22%2C%22value%22%3A%5B%22liver%20and%20intrahepatic%20bile%20ducts%22%5D%7D%2C%22op%22%3A%22in%22%7D%2C%7B%22op%22%3A%22in%22%2C%22content%22%3A%7B%22field%22%3A%22cases.project.program.name%22%2C%22value%22%3A%5B%22TCGA%22%5D%7D%7D%2C%7B%22op%22%3A%22in%22%2C%22content%22%3A%7B%22field%22%3A%22cases.project.project_id%22%2C%22value%22%3A%5B%22TCGA-LIHC%22%5D%7D%7D%5D%7D)  TCGA-CHOL | Liver hepatocellular carcinoma | Primary tumor | [adenomas and adenocarcinomas](https://portal.gdc.cancer.gov/exploration?filters=%7B%22op%22%3A%22and%22%2C%22content%22%3A%5B%7B%22content%22%3A%7B%22field%22%3A%22cases.diagnoses.tissue_or_organ_of_origin%22%2C%22value%22%3A%5B%22intrahepatic%20bile%20duct%22%2C%22liver%22%5D%7D%2C%22op%22%3A%22in%22%7D%2C%7B%22op%22%3A%22in%22%2C%22content%22%3A%7B%22field%22%3A%22cases.disease_type%22%2C%22value%22%3A%5B%22adenomas%20and%20adenocarcinomas%22%5D%7D%7D%2C%7B%22content%22%3A%7B%22field%22%3A%22cases.primary_site%22%2C%22value%22%3A%5B%22liver%20and%20intrahepatic%20bile%20ducts%22%5D%7D%2C%22op%22%3A%22in%22%7D%2C%7B%22op%22%3A%22in%22%2C%22content%22%3A%7B%22field%22%3A%22cases.project.program.name%22%2C%22value%22%3A%5B%22TCGA%22%5D%7D%7D%2C%7B%22op%22%3A%22in%22%2C%22content%22%3A%7B%22field%22%3A%22cases.samples.sample_type%22%2C%22value%22%3A%5B%22primary%20tumor%22%5D%7D%7D%5D%7D) |
| ***Lung*** | [TCGA-LUAD](https://portal.gdc.cancer.gov/exploration?filters=%7B%22op%22%3A%22and%22%2C%22content%22%3A%5B%7B%22content%22%3A%7B%22field%22%3A%22cases.diagnoses.tissue_or_organ_of_origin%22%2C%22value%22%3A%5B%22lower%20lobe%2C%20lung%22%2C%22lung%2C%20nos%22%2C%22main%20bronchus%22%2C%22middle%20lobe%2C%20lung%22%2C%22overlapping%20lesion%20of%20lung%22%2C%22upper%20lobe%2C%20lung%22%5D%7D%2C%22op%22%3A%22in%22%7D%2C%7B%22content%22%3A%7B%22field%22%3A%22cases.primary_site%22%2C%22value%22%3A%5B%22bronchus%20and%20lung%22%5D%7D%2C%22op%22%3A%22in%22%7D%2C%7B%22op%22%3A%22in%22%2C%22content%22%3A%7B%22field%22%3A%22cases.project.program.name%22%2C%22value%22%3A%5B%22TCGA%22%5D%7D%7D%2C%7B%22op%22%3A%22in%22%2C%22content%22%3A%7B%22field%22%3A%22cases.project.project_id%22%2C%22value%22%3A%5B%22TCGA-LUAD%22%5D%7D%7D%5D%7D)  [TCGA-LUSC](https://portal.gdc.cancer.gov/exploration?filters=%7B%22op%22%3A%22and%22%2C%22content%22%3A%5B%7B%22content%22%3A%7B%22field%22%3A%22cases.diagnoses.tissue_or_organ_of_origin%22%2C%22value%22%3A%5B%22lower%20lobe%2C%20lung%22%2C%22lung%2C%20nos%22%2C%22main%20bronchus%22%2C%22middle%20lobe%2C%20lung%22%2C%22overlapping%20lesion%20of%20lung%22%2C%22upper%20lobe%2C%20lung%22%5D%7D%2C%22op%22%3A%22in%22%7D%2C%7B%22content%22%3A%7B%22field%22%3A%22cases.primary_site%22%2C%22value%22%3A%5B%22bronchus%20and%20lung%22%5D%7D%2C%22op%22%3A%22in%22%7D%2C%7B%22op%22%3A%22in%22%2C%22content%22%3A%7B%22field%22%3A%22cases.project.program.name%22%2C%22value%22%3A%5B%22TCGA%22%5D%7D%7D%2C%7B%22op%22%3A%22in%22%2C%22content%22%3A%7B%22field%22%3A%22cases.project.project_id%22%2C%22value%22%3A%5B%22TCGA-LUSC%22%5D%7D%7D%5D%7D) | Lung adenocarcinoma  Lung squamous cell carcinoma | Primary tumor | [cystic, mucinous, and serous neoplasms](https://portal.gdc.cancer.gov/exploration?filters=%7B%22op%22%3A%22and%22%2C%22content%22%3A%5B%7B%22content%22%3A%7B%22field%22%3A%22cases.diagnoses.tissue_or_organ_of_origin%22%2C%22value%22%3A%5B%22lower%20lobe%2C%20lung%22%2C%22lung%2C%20nos%22%2C%22main%20bronchus%22%2C%22middle%20lobe%2C%20lung%22%2C%22overlapping%20lesion%20of%20lung%22%2C%22upper%20lobe%2C%20lung%22%5D%7D%2C%22op%22%3A%22in%22%7D%2C%7B%22op%22%3A%22in%22%2C%22content%22%3A%7B%22field%22%3A%22cases.disease_type%22%2C%22value%22%3A%5B%22cystic%2C%20mucinous%20and%20serous%20neoplasms%22%5D%7D%7D%2C%7B%22content%22%3A%7B%22field%22%3A%22cases.primary_site%22%2C%22value%22%3A%5B%22bronchus%20and%20lung%22%5D%7D%2C%22op%22%3A%22in%22%7D%2C%7B%22op%22%3A%22in%22%2C%22content%22%3A%7B%22field%22%3A%22cases.project.program.name%22%2C%22value%22%3A%5B%22TCGA%22%5D%7D%7D%2C%7B%22op%22%3A%22in%22%2C%22content%22%3A%7B%22field%22%3A%22cases.samples.sample_type%22%2C%22value%22%3A%5B%22primary%20tumor%22%5D%7D%7D%5D%7D) |
| ***Non-Hodgkin’s lymphoma*** | [TCGA-DLBC](https://portal.gdc.cancer.gov/exploration?filters=%7B%22op%22%3A%22and%22%2C%22content%22%3A%5B%7B%22content%22%3A%7B%22field%22%3A%22cases.diagnoses.tissue_or_organ_of_origin%22%2C%22value%22%3A%5B%22intra-abdominal%20lymph%20nodes%22%2C%22intrathoracic%20lymph%20nodes%22%2C%22lymph%20node%2C%20nos%22%2C%22lymph%20nodes%20of%20axilla%20or%20arm%22%2C%22lymph%20nodes%20of%20head%2C%20face%20and%20neck%22%2C%22lymph%20nodes%20of%20inguinal%20region%20or%20leg%22%2C%22lymph%20nodes%20of%20multiple%20regions%22%2C%22pelvic%20lymph%20nodes%22%5D%7D%2C%22op%22%3A%22in%22%7D%2C%7B%22content%22%3A%7B%22field%22%3A%22cases.primary_site%22%2C%22value%22%3A%5B%22lymph%20nodes%22%5D%7D%2C%22op%22%3A%22in%22%7D%2C%7B%22op%22%3A%22in%22%2C%22content%22%3A%7B%22field%22%3A%22cases.project.program.name%22%2C%22value%22%3A%5B%22TCGA%22%5D%7D%7D%2C%7B%22op%22%3A%22in%22%2C%22content%22%3A%7B%22field%22%3A%22cases.project.project_id%22%2C%22value%22%3A%5B%22TCGA-DLBC%22%5D%7D%7D%2C%7B%22op%22%3A%22in%22%2C%22content%22%3A%7B%22field%22%3A%22cases.samples.sample_type%22%2C%22value%22%3A%5B%22primary%20tumor%22%5D%7D%7D%5D%7D) | Lymphoid Neoplasm Diffuse Large B-cell Lymphoma | Primary tumor | [mature b-cell lymphomas](https://portal.gdc.cancer.gov/exploration?filters=%7B%22op%22%3A%22and%22%2C%22content%22%3A%5B%7B%22content%22%3A%7B%22field%22%3A%22cases.diagnoses.tissue_or_organ_of_origin%22%2C%22value%22%3A%5B%22intra-abdominal%20lymph%20nodes%22%2C%22intrathoracic%20lymph%20nodes%22%2C%22lymph%20node%2C%20nos%22%2C%22lymph%20nodes%20of%20axilla%20or%20arm%22%2C%22lymph%20nodes%20of%20head%2C%20face%20and%20neck%22%2C%22lymph%20nodes%20of%20inguinal%20region%20or%20leg%22%2C%22lymph%20nodes%20of%20multiple%20regions%22%2C%22pelvic%20lymph%20nodes%22%5D%7D%2C%22op%22%3A%22in%22%7D%2C%7B%22content%22%3A%7B%22field%22%3A%22cases.primary_site%22%2C%22value%22%3A%5B%22lymph%20nodes%22%5D%7D%2C%22op%22%3A%22in%22%7D%2C%7B%22op%22%3A%22in%22%2C%22content%22%3A%7B%22field%22%3A%22cases.project.program.name%22%2C%22value%22%3A%5B%22TCGA%22%5D%7D%7D%2C%7B%22op%22%3A%22in%22%2C%22content%22%3A%7B%22field%22%3A%22cases.samples.sample_type%22%2C%22value%22%3A%5B%22primary%20tumor%22%5D%7D%7D%5D%7D) |
| ***Rectal*** | [TCGA-READ](https://portal.gdc.cancer.gov/exploration?filters=%7B%22op%22%3A%22and%22%2C%22content%22%3A%5B%7B%22content%22%3A%7B%22field%22%3A%22cases.diagnoses.tissue_or_organ_of_origin%22%2C%22value%22%3A%5B%22appendix%22%2C%22ascending%20colon%22%2C%22cecum%22%2C%22colon%2C%20nos%22%2C%22descending%20colon%22%2C%22hepatic%20flexure%20of%20colon%22%2C%22overlapping%20lesion%20of%20colon%22%2C%22rectosigmoid%20junction%22%2C%22rectum%2C%20nos%22%2C%22sigmoid%20colon%22%2C%22splenic%20flexure%20of%20colon%22%2C%22transverse%20colon%22%5D%7D%2C%22op%22%3A%22in%22%7D%2C%7B%22content%22%3A%7B%22field%22%3A%22cases.primary_site%22%2C%22value%22%3A%5B%22colon%22%2C%22rectosigmoid%20junction%22%2C%22rectum%22%5D%7D%2C%22op%22%3A%22in%22%7D%2C%7B%22op%22%3A%22in%22%2C%22content%22%3A%7B%22field%22%3A%22cases.project.program.name%22%2C%22value%22%3A%5B%22TCGA%22%5D%7D%7D%2C%7B%22op%22%3A%22in%22%2C%22content%22%3A%7B%22field%22%3A%22cases.project.project_id%22%2C%22value%22%3A%5B%22TCGA-READ%22%5D%7D%7D%5D%7D) | Rectum adenocarcinoma | Primary tumor | [adenomas and adenocarcinomas](https://portal.gdc.cancer.gov/exploration?filters=%7B%22op%22%3A%22and%22%2C%22content%22%3A%5B%7B%22content%22%3A%7B%22field%22%3A%22cases.diagnoses.tissue_or_organ_of_origin%22%2C%22value%22%3A%5B%22appendix%22%2C%22ascending%20colon%22%2C%22cecum%22%2C%22colon%2C%20nos%22%2C%22descending%20colon%22%2C%22hepatic%20flexure%20of%20colon%22%2C%22overlapping%20lesion%20of%20colon%22%2C%22rectosigmoid%20junction%22%2C%22rectum%2C%20nos%22%2C%22sigmoid%20colon%22%2C%22splenic%20flexure%20of%20colon%22%2C%22transverse%20colon%22%5D%7D%2C%22op%22%3A%22in%22%7D%2C%7B%22op%22%3A%22in%22%2C%22content%22%3A%7B%22field%22%3A%22cases.primary_site%22%2C%22value%22%3A%5B%22rectum%22%5D%7D%7D%2C%7B%22op%22%3A%22in%22%2C%22content%22%3A%7B%22field%22%3A%22cases.project.program.name%22%2C%22value%22%3A%5B%22TCGA%22%5D%7D%7D%2C%7B%22op%22%3A%22in%22%2C%22content%22%3A%7B%22field%22%3A%22cases.samples.sample_type%22%2C%22value%22%3A%5B%22primary%20tumor%22%5D%7D%7D%5D%7D) |
| ***Thyroid*** | [TCGA-THCA](https://portal.gdc.cancer.gov/exploration?filters=%7B%22op%22%3A%22and%22%2C%22content%22%3A%5B%7B%22content%22%3A%7B%22field%22%3A%22cases.diagnoses.tissue_or_organ_of_origin%22%2C%22value%22%3A%5B%22thyroid%20gland%22%5D%7D%2C%22op%22%3A%22in%22%7D%2C%7B%22content%22%3A%7B%22field%22%3A%22cases.primary_site%22%2C%22value%22%3A%5B%22thyroid%20gland%22%5D%7D%2C%22op%22%3A%22in%22%7D%2C%7B%22op%22%3A%22in%22%2C%22content%22%3A%7B%22field%22%3A%22cases.project.program.name%22%2C%22value%22%3A%5B%22TCGA%22%5D%7D%7D%2C%7B%22op%22%3A%22in%22%2C%22content%22%3A%7B%22field%22%3A%22cases.project.project_id%22%2C%22value%22%3A%5B%22TCGA-THCA%22%5D%7D%7D%5D%7D) | Thyroid carcinoma | Primary tumor | [adenomas and adenocarcinomas](https://portal.gdc.cancer.gov/exploration?filters=%7B%22op%22%3A%22and%22%2C%22content%22%3A%5B%7B%22content%22%3A%7B%22field%22%3A%22cases.diagnoses.tissue_or_organ_of_origin%22%2C%22value%22%3A%5B%22thyroid%20gland%22%5D%7D%2C%22op%22%3A%22in%22%7D%2C%7B%22op%22%3A%22in%22%2C%22content%22%3A%7B%22field%22%3A%22cases.disease_type%22%2C%22value%22%3A%5B%22adenomas%20and%20adenocarcinomas%22%5D%7D%7D%2C%7B%22content%22%3A%7B%22field%22%3A%22cases.primary_site%22%2C%22value%22%3A%5B%22thyroid%20gland%22%5D%7D%2C%22op%22%3A%22in%22%7D%2C%7B%22op%22%3A%22in%22%2C%22content%22%3A%7B%22field%22%3A%22cases.project.program.name%22%2C%22value%22%3A%5B%22TCGA%22%5D%7D%7D%2C%7B%22op%22%3A%22in%22%2C%22content%22%3A%7B%22field%22%3A%22cases.samples.sample_type%22%2C%22value%22%3A%5B%22primary%20tumor%22%5D%7D%7D%5D%7D) |

**Supplementary Section 2: Prediction Performance of OncoRTT using three classifiers for the three sets of features**

We evaluated OncoRTT in terms of AUC for each cancer type using two ML classifiers (RF and XGBoost) and one DL classifier (DNN) for different sets of FVs: Integrated-based Embedding-based and Omics-based FVs. This process allowed us to test our methods' performance in multiple experiments before selecting the model with the best-obtained results reported in the main manuscript. Supplementary Table S2 provides the results for three classifiers using three sets of features separately for ten cancer types. As we can see from this figure, the best-obtained results were achieved when using DNN with the integrated-based features. Thus, we selected this DL model that achieved the highest AUC for our final model to identify therapeutic targets. In addition, based on this figure, we can see a clear trend in the results that using DNN outperformed RF and XGBoost when using Integrated-based FV and Embedding-based FV for all cancer types.

​**Supplementary Table S2.** Comparing the prediction performances of the classifiers per cancer, in terms of AUC, using three different sets of features.

***Labels:*** *Among all classifiers and all FV sets: Bold underline indicates the best performances, and only bold indicates the second-best. Within each FV set, to compare the three classifiers, the green highlight indicates the best performances.*

| **Cancer**  **type** | **Integrated-based FV** | | | | **Embed-based FV** | | | | | **Omics-based FV** | | | |
| --- | --- | --- | --- | --- | --- | --- | --- | --- | --- | --- | --- | --- | --- |
|  | **DNN** | **XGBoost** | **RF** | **SVM** | | **DNN** | **XGBoost** | **RF** | **SVM** | **DNN** | **XGBoost** | **RF** | **SVM** |
| ***Bladder*** | **0.92** | 0.84 | 0.73 | 0.70 | | **0.91** | 0.71 | 0.83 | 0.70 | 0.69 | 0.84 | 0.81 | 0.71 |
| ***Breast*** | **0.94** | 0.80 | 0.76 | 0.76 | | **0.89** | 0.71 | 0.78 | 0.75 | 0.72 | 0.78 | 0.77 | 0.70 |
| ***Colon*** | **0.93** | 0.75 | 0.73 | 0.75 | | **0.86** | 0.74 | 0.64 | 0.72 | 0.63 | 0.66 | 0.67 | 0.60 |
| ***Kidney*** | **0.94** | 0.69 | 0.65 | 0.64 | | **0.88** | 0.57 | 0.73 | 0.60 | 0.76 | 0.74 | 0.72 | 0.69 |
| ***Leukemia*** | **0.95** | 0.77 | 0.70 | 0.70 | | **0.88** | 0.65 | 0.82 | 0.67 | 0.77 | 0.81 | 0.80 | 0.72 |
| ***Liver*** | **0.88** | 0.77 | 0.68 | 0.63 | | **0.82** | 0.65 | 0.80 | 0.63 | 0.75 | 0.82 | 0.82 | 0.41 |
| ***Lung*** | **0.94** | 0.83 | 0.73 | 0.70 | | **0.86** | 0.72 | 0.79 | 0.70 | 0.73 | 0.78 | 0.81 | 0.78 |
| ***Non-Hodgkin’s*** | **0.92** | 0.79 | 0.72 | 0.69 | | **0.87** | 0.66 | 0.74 | 0.67 | 0.77 | 0.74 | 0.76 | 0.75 |
| ***Rectal*** | **0.90** | 0.60 | 0.71 | 0.69 | | **0.87** | 0.60 | 0.63 | 0.69 | 0.61 | 0.62 | 0.62 | 0.49 |
| ***Thyroid*** | **0.92** | 0.74 | 0.75 | 0.75 | | **0.88** | 0.69 | 0.74 | 0.75 | 0.78 | 0.74 | 0.78 | 0.76 |

### **Supplementary Table S3:** Top 10-ranked novel predicted therapeutic targets for **bladder** cancer with evidence linking the targets and disease.

| **Gene** | **UniProt ID** | **Prediction Score** | **Protein Name** | **# of evidence** | **Association type** |
| --- | --- | --- | --- | --- | --- |
| ***LCE3D*** | Q9BYE3 | 0.9707 | Late Cornified Envelope 3D | 1 | Pathways |
| ***FAM133B*** | Q5BKY9 | 0.9668 | Family With Sequence Similarity 133 Member B | 1 | Text mining |
| ***NEGR1*** | Q7Z3B1 | 0.9650 | Neuronal Growth Regulator 1 | 1 | Text mining |
| ***SERPINB4*** | P48594 | 0.9624 | Serpin Family B Member 4 | 0 | Linked to other cancer |
| ***CLEC4D*** | Q8WXI8 | 0.9616 | C-Type Lectin Domain Family 4 Member D | 0 | Linked to other related cancers |
| ***BTLA*** | Q7Z6A9 | 0.9603 | B And T Lymphocyte Associated | 2 | Text mining |
| ***HMGB1*** | P09429 | 0.9600 | High Mobility Group Box 1 | 36 | Text mining |
| ***CEACAM1*** | P13688 | 0.9586 | GTP Binding Protein 10 | 10 | Text mining |
| ***GTPBP10*** | A4D1E9 | 0.9573 | RAB12, Member RAS Oncogene Family | 0 | Linked to other related cancers |
| ***RAB12*** | Q6IQ22 | 0.9566 | RAB12, Member RAS Oncogene Family | 0 | Linked to other cancer |

### **Supplementary Table S4:** Top 10-ranked novel predicted therapeutic targets for **breast** cancer with evidence linking the targets and disease.

| **Gene** | **UniProt ID** | **Prediction Score** | **Protein Name** | **# of evidence** | **Association type** |
| --- | --- | --- | --- | --- | --- |
| ***COL1A2*** | P08123 | 0.9866 | Collagen Type I Alpha 2 Chain | 19 | Text mining, RNA expression, Pathways, Genetic association |
| ***ANAPC15*** | P60006 | 0.9851 | Anaphase Promoting Complex Subunit 15 | 0 | Linked to [endometrial cancer](https://platform.opentargets.org/disease/MONDO_0011962) |
| ***SPP1*** | P10451 | 0.9836 | Secreted Phosphoprotein 1 | 93 | Text mining, RNA expression, Pathways |
| ***PPP1R7*** | Q15435 | 0.9759 | Protein Phosphatase 1 Regulatory Subunit 7 | 1 | Text mining |
| ***GAS1*** | P54826 | 0.9717 | Growth Arrest Specific 1 | 12 | Text mining, RNA expression |
| ***TUSC2*** | O75896 | 0.9677 | Tumor Suppressor 2, Mitochondrial Calcium Regulator | 2 | Text mining |
| ***FGA*** | P02671 | 0.9650 | Fibrinogen Alpha Chain | 26 | Text mining, RNA expression, Pathways |
| ***FOXD1*** | Q16676 | 0.9645 | Forkhead Box D1 | 1 | Genetic association |
| ***HMGN2*** | P05204 | 0.9644 | High Mobility Group Nucleosomal Binding Domain 2 | 0 | Linked to other cancers |
| ***C1QB*** | P02746 | 0.9602 | Complement C1q B Chain | 2 | Text mining, RNA expression |

### **Supplementary Table S5:** Top 10-ranked novel predicted therapeutic targets for **leukemia** cancer with evidence linking the targets and disease.

| **Gene** | **UniProt ID** | **Prediction Score** | **Protein Name** | **# of evidence** | **Association type** |
| --- | --- | --- | --- | --- | --- |
| ***MDGA1*** | [Q8NFP4](http://www.uniprot.org/uniprot/Q8NFP4) | 0.9943 | MAM Domain Containing Glycosylphosphatidylinositol Anchor 1 | 1 | Somatic mutations |
| ***MYBPC3*** | [Q14896](http://www.uniprot.org/uniprot/Q14896) | 0.9940 | Myosin Binding Protein C3 | 3 | Text mining |
| ***CREB1*** | [P16220](http://www.uniprot.org/uniprot/P16220) | 0.9934 | CAMP Responsive Element Binding Protein 1 | 107 | Somatic mutations, pathways, Text mining |
| ***CHL1*** | O00533 | 0.9923 | Cell Adhesion Molecule L1 Like | 44 | Text mining |
| ***CAMKK2*** | Q96RR4 | 0.9909 | Calcium/Calmodulin Dependent Protein Kinase Kinase 2 | 0 | Linked to other cancers |
| ***COL1A1*** | P02452 | 0.9896 | Collagen Type I Alpha 1 Chain | 7 | Somatic mutations, Text mining |
| ***CAMK4*** | Q16566 | 0.9876 | Calcium/Calmodulin Dependent Protein Kinase IV | 6 | Genetic associations, Text mining |
| ***EPHB1*** | P54762 | 0.9867 | EPH Receptor B1 | 4 | Drugs, Text mining |
| ***MAST2*** | Q6P0Q8 | 0.9851 | Microtubule Associated Serine/Threonine Kinase 2 | 1 | Text mining |
| ***NFRKB*** | Q6P4R8 | 0.9831 | Nuclear Factor Related To KappaB Binding Protein | 1 | Text mining |

### **Supplementary Table S6:** Top 10-ranked novel predicted therapeutic targets for **liver** cancer with evidence linking the targets and disease. Linked to liver cancer or [hepatocellular carcinoma](https://platform.opentargets.org/disease/EFO_0000182), the most common type of primary liver cancer

| **Gene** | **UniProt ID** | **Prediction Score** | **Protein Name** | **# of evidence** | **Association type** |
| --- | --- | --- | --- | --- | --- |
| ***MT1E*** | P04732 | 0.9362 | Metallothionein 1E | 6 | Text mining, RNA expression |
| ***SRSF4*** | Q08170 | 0.8946 | Serine And Arginine Rich Splicing Factor 4 | 6 | Text mining |
| ***RUVBL1*** | Q9Y265 | 0.8816 | RuvB Like AAA ATPase 1 | 0 | Linked Prostate and other cancer |
| ***PTGER4*** | P35408 | 0.8570 | Prostaglandin E2 receptor EP4 subtype | 14 | Text mining, RNA expression |
| ***PKMYT1*** | Q99640 | 0.7676 | Prostaglandin E Receptor 4 | 12 | Text mining, RNA expression |
| ***MUSK*** | O15146 | 0.7335 | Muscle Associated Receptor Tyrosine Kinase | 1 | Text mining |
| ***COL11A2*** | P13942 | 0.7287 | Collagen Type XI Alpha 2 Chain | 0 | Linked other cancers |
| ***EPHA1*** | P21709 | 0.7145 | Erythropoietin-Producing Hepatoma Receptor | 13 | Text mining |
| ***SPINK1*** | P00995 | 0.6917 | Serine Peptidase Inhibitor Kazal Type 1 | 37 | Text mining, RNA expression |
| ***ANKRD22*** | Q5VYY1 | 0.6902 | Ankyrin Repeat Domain 22 | 0 | Linked other cancers |
